# Alternative transcription cycle for bacterial RNA polymerase

**DOI:** 10.1038/s41467-019-14208-9

**Published:** 2020-01-23

**Authors:** Timothy T. Harden, Karina S. Herlambang, Mathew Chamberlain, Jean-Benoît Lalanne, Christopher D. Wells, Gene-Wei Li, Robert Landick, Ann Hochschild, Jane Kondev, Jeff Gelles

**Affiliations:** 1 Department of Physics, Brandeis University, Waltham, MA 02454 USA; 2 Department of Biochemistry, Brandeis University, Waltham, MA 02454 USA; 3 Department of Biology, Massachusetts Institute of Technology, Cambridge, MA 02139 USA; 4 Department of Physics, Massachusetts Institute of Technology, Cambridge, MA 02139 USA; 5 Department of Microbiology, Blavatnick Institute, Harvard Medical School, Boston, MA 02115 USA; 6 Department of Biochemistry and Department of Bacteriology, University of Wisconsin, Madison, WI 53706 USA

**Keywords:** Single-molecule biophysics, Transcription

## Abstract

RNA polymerases (RNAPs) transcribe genes through a cycle of recruitment to promoter DNA, initiation, elongation, and termination. After termination, RNAP is thought to initiate the next round of transcription by detaching from DNA and rebinding a new promoter. Here we use single-molecule fluorescence microscopy to observe individual RNAP molecules after transcript release at a terminator. Following termination, RNAP almost always remains bound to DNA and sometimes exhibits one-dimensional sliding over thousands of basepairs. Unexpectedly, the DNA-bound RNAP often restarts transcription, usually in reverse direction, thus producing an antisense transcript. Furthermore, we report evidence of this secondary initiation in live cells, using genome-wide RNA sequencing. These findings reveal an alternative transcription cycle that allows RNAP to reinitiate without dissociating from DNA, which is likely to have important implications for gene regulation.

## Abstract (web summary)

In the canonical bacterial transcription, both nascent transcript and polymerase dissociate from template DNA. By employing multi-color single-molecule fluorescence imaging, here the authors show that RNA polymerases remain bound to DNA after the transcript release.

## Introduction

In all organisms, gene transcription is usually viewed as initiating with the binding, assisted by accessory transcription factor proteins^1,2^, of a RNA polymerase (RNAP) molecule from solution to a transcription promoter. In bacteria, the core RNAP first associates with an initiation factor, a sigma subunit, which confers the ability to recognize promoter DNA and initiate RNA synthesis^3^. In the canonical bacterial transcription cycle, transcript synthesis concludes when release of the nascent RNA molecule from the polymerase is triggered by specific DNA sequences (intrinsic terminators) or by termination factors (e.g., the *E. coli* Rho protein)^4^. While some studies suggest that RNAP dissociates rapidly from DNA upon intrinsic transcription termination, others suggest that a long-lived RNAP-DNA complex can persist after termination^5–8^.

Antisense transcription, which produces RNAs that have sequences at least in part complementary to ordinary sense gene transcripts, has been observed in organisms from bacteria to humans and is typically initiated from locations throughout the entire genome^9–12^. While the global biological significance of this pervasive antisense transcription has been questioned^13,14^, antisense RNA production has demonstrated roles in regulating expression of many individual sense genes^15–22^. The origins of antisense transcripts are incompletely understood. However, the relevant genetic elements, molecular mechanisms, and regulatory machinery are being explored (e.g., refs. ^23–25^).

In this study, we use single-molecule fluorescence microscopy in vitro to observe transcript production by bacterial RNAP and, significantly, also to follow the fate of the RNAP molecule after intrinsic termination of transcription. Under conditions designed to mimic the ionic composition of bacterial cytoplasm, RNAP most often does not follow the canonical transcription cycle in which each recruitment event of a polymerase molecule to DNA can produce at most only one molecule of RNA primary transcript. Instead, the experiments reveal a frequently occurring alternative transcription cycle through which, as a consequence of its recruitment to a promoter, a single RNAP molecule can produce multiple transcripts, including transcripts that are antisense to the first RNA molecule produced. In addition, we show evidence from end-enhanced genome-wide RNA sequencing suggesting that the alternative cycle is a widespread mechanism for synthesis of antisense transcripts in bacteria.

## Results

### Observing RNAP molecules from initiation through termination

**Fig. 1:**
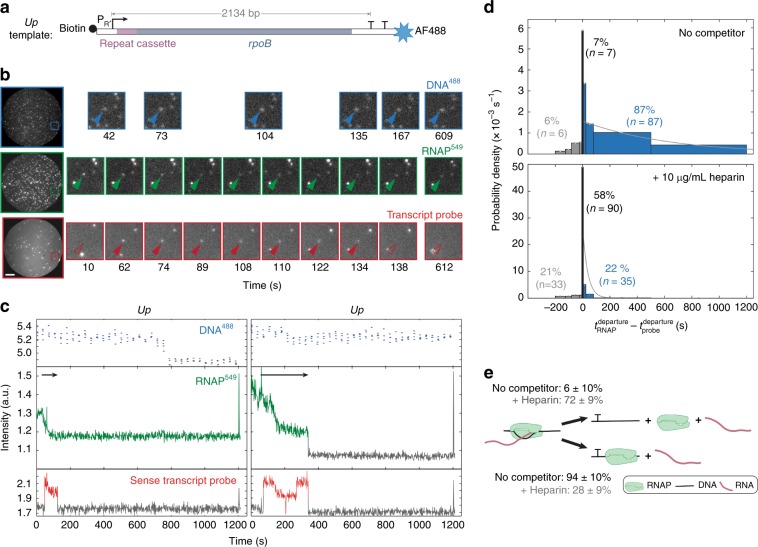
RNAP usually remains bound to the DNA template following transcript release at an intrinsic terminator. **a** *Up* transcription template. The template contains a wild type λ P_R´_ promoter region (bent arrow) followed by seven tandem repeats of a 21 bp cassette (maroon), a partial sequence of *E. coli rpoB* coding region (gray) and two consecutive intrinsic terminators (T): λ T_Rʹ_ and T7 T_E_, which have termination efficiencies in vitro of 93-95% and 88 ± 2%, respectively^59–61^. Biotin is positioned upstream (“*up*”) of promoter so that RNAP moves away from the streptavidin-coated slide during transcription. **b** Left: Images (65 × 65 µm) of the same microscope field of view of DNA^488^ (blue), RNAP^549^ (green) and Cy5-transcript hybridization probe (red). Right: magnified views of the marked regions at various times during the experiment; NTPs were introduced at time *t* ~ −10 s. Blue arrows mark the location of a DNA spot, green and red arrows mark the surface location in the other images, with presence (filled arrows) and absence (open arrows) of a co-localized fluorescence spot indicated. Scale bar, 10 µm. **c** Example fluorescence emission records from the locations of two DNA spots from the same experiment. Gray color marks intervals during which no fluorescent spot was seen. Arrows mark intervals of transcript elongation. Left: RNAP remains after probe departs (data from marked molecule in (**b**)) Right: RNAP and probe depart simultaneously. **d** Normalized histogram of RNAP^549^ departure time relative to Cy5-transcript probe departure from the same DNA spot for elongation reactions in the absence and presence of heparin. RNAP^549^ spot departed either before (gray), within 4 s of (black), or after (blue) transcript probe spot departure. The 4 s threshold was chosen because it is the maximum interval between consecutive frames. Gray curves are single exponential fits to the RNAP departure times following probe departure (see the “Methods” section). **e** Reaction scheme indicating the fraction of terminating ECs for which RNAP^549^ retains association with DNA after termination, calculated from the data in (**d**).

To examine the behavior of individual molecules of RNAP after transcription termination, we used a previously developed single-molecule fluorescence technique to study single-round transcription. In brief, we tethered fluorescent DNA molecules (DNA^488^) containing a promoter sequence, a 2.1 kbp transcription unit, and two consecutive intrinsic terminators to the surface of a glass flow chamber (Fig. 1a). We incubated the chamber with a solution containing σ^70^ holoenzyme made with core RNAP fluorescently labeled with a BG-549 dye on a SNAP tag on the carboxyl-terminal end of the beta subunit (RNAP^549^). Following open complex formation (Fig. 1b, left), we initiated transcription by introducing 0.5 mM each ATP, CTP, GTP, and UTP at time *t* = 0, along with a Cy5-labeled oligonucleotide probe. The probe detects nascent transcript by hybridization to a repeat target sequence near the 5′ end of the RNA (Fig. 1a). Analogously to previous experiments with labeled σ subunits^26,27^, we observed the appearance of probe fluorescence spots that colocalized with RNAP^549^ and DNA^488^ spots, reflecting the hybridization of probe with the nascent RNA in individual transcription elongation complexes (ECs; e.g., *t* = 62 s in Fig. 1b, c, left). The probe spot typically later disappeared (e.g., *t* = 138 s in Fig. 1b, c, left); this disappearance was scored as transcription termination since RNA release at intrinsic terminators is rapid^8^ and the lifetime of transcript probe is not significantly reduced by photobleaching under these conditions (Supplementary Fig. 1A).

### RNAP almost always remains bound to DNA after termination

In two replicate experiments, we observed a total of 100 molecules in which core RNAP^549^ fluorescence was visible when the probe spot appeared. Of these, 94% retained RNAP^549^ spots at the time of termination indicated by transcript release. Consistent with our earlier study with labeled σ^70^, most (87/100) RNAP^549^ molecules did not dissociate upon termination (Fig. 1d, top). Instead, most persisted after transcript departure and eventually dissociated with a mean lifetime of 1140 ± 240 s (after accounting for photobleaching; see the “Methods” section and Supplementary Fig. 1B). Thus, nearly all RNAP that terminates under the conditions of these experiments stays associated with DNA after transcript release at an intrinsic terminator (Fig. 1e). We previously showed that σ^70^-containing ECs behave similarly: on this same template 21% of ECs reached the terminator with bound σ^70^ and in the majority (74%) of these σ^70^ remained associated with DNA after termination^26^. Taken together these data imply that both RNAP and σ^70^RNAP persist on DNA after transcription termination, usually for hundreds of seconds.

The presence of long-lived, DNA-RNAP complexes after termination is surprising. Heparin is a polyanion that can disrupt early promoter DNA-RNAP complexes in the initiation pathway^28^. When we added 10 µg mL^−1^ heparin together with the NTPs, we still observed transcript production from the open complexes as expected, but now most RNAP molecules dissociated from DNA within 4 s of transcript departure (Fig. 1d, bottom). Those that did persist showed a characteristic lifetime (38 ± 10 s) greatly reduced relative to that in the absence of heparin. The observation that a polyanion competitor can dramatically reduce retention of RNAP on DNA after termination suggests that the retained RNAP interacts primarily with the DNA backbone without the more extensive contacts with DNA bases that occur in open complexes and ECs.

### RNAP can diffuse along DNA after termination

In our single-molecule experiments, transcript release was almost invariably preceded by a gradual decrease in RNAP^549^ fluorescence intensity (Fig. 1c, black arrows). This decrease is expected, because during transcript elongation RNAP moves along the DNA so that its time-averaged distance from the chamber surface increases, decreasing the intensity of TIRF excitation and leading to reduced emission. A systematic increase in spot width was also observed, consistent with the idea that the intensity changes are due to net translocation of the elongation complex along DNA and result in increased Brownian motion of the DNA-tethered RNAP^549^ (Supplementary Fig. 1C, D, and E)^29^. In an experiment with inverted DNA (i.e., with the biotin tag placed at the downstream end of the DNA), we instead observed increasing RNAP^549^ fluorescence during transcript probe co-localization, as predicted (Supplementary Fig. 1F).

**Fig. 2:**
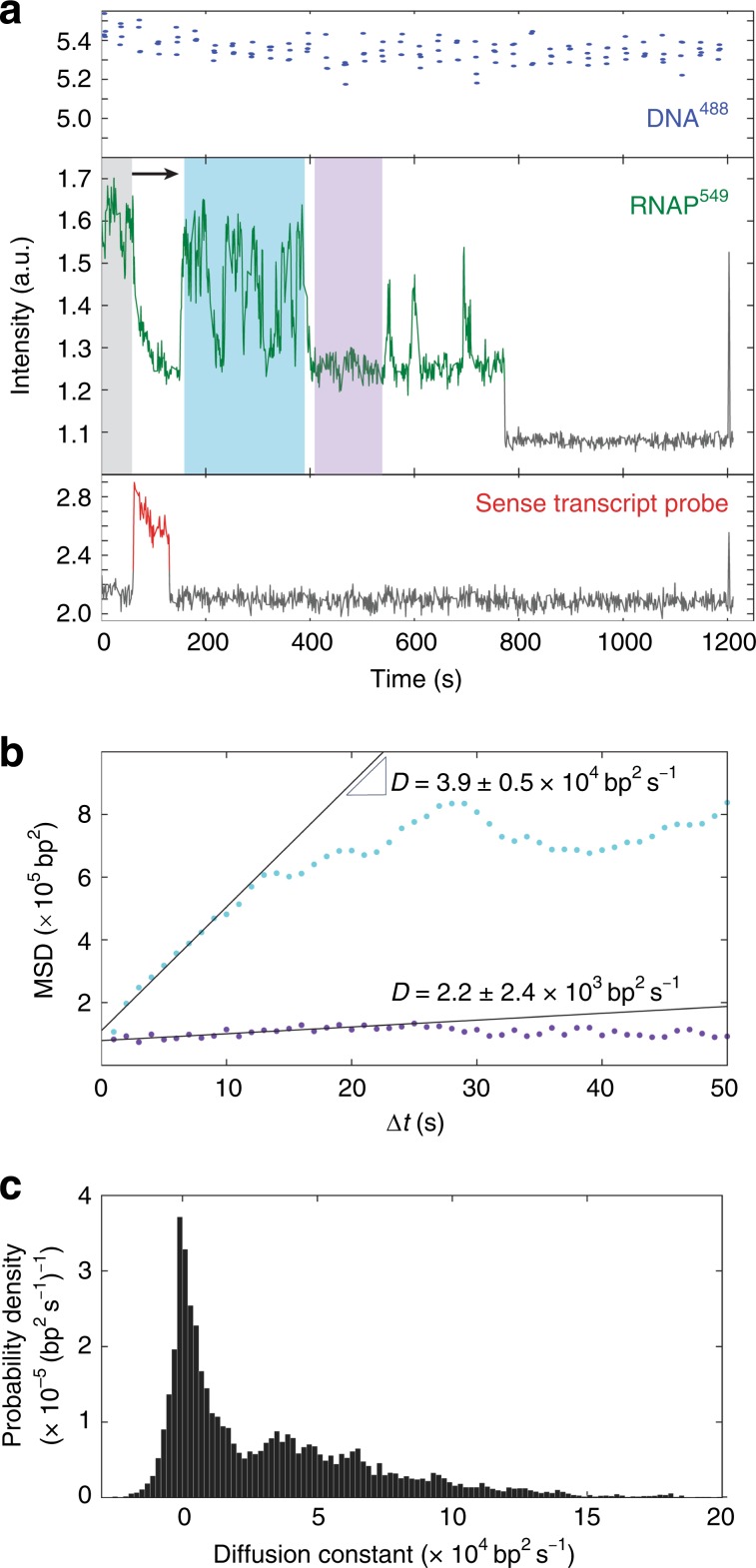
RNAP can diffuse along DNA after termination. **a** A single-molecule emission record, as in Fig. 1c, for a different DNA spot location. Gray, teal, and purple highlight time intervals of high RNAP^549^ fluorescence before detection of transcript probe and post-termination intervals of fluctuating and low fluorescence, respectively. **b** Mean squared displacement (MSD) of RNAP^549^ position on DNA during the teal and purple intervals in (**a**). Linear fits to the first ten points of each MSD curve yield the effective diffusion coefficients over 10 s intervals, *D*. **c** Normalized histogram of *D* values measured separately for every 50 s window in *n* *=* 41 recordings of RNAP^549^ retained on DNA after termination (13,522 windows total).

In contrast to the gradual decrease in RNAP^549^ fluorescence observed before transcript release, we saw a different behavior after termination. After transcript release, we often (in roughly half of the Fig. 1d blue population) saw episodes of rapid, bidirectional fluctuation in RNAP^549^ intensity (Fig. 2a and Supplementary Fig. 2A–I, teal). No correlated fluctuation was seen in DNA template fluorescence (e.g., Fig. 2a, top), suggesting that the RNAP^549^ intensity fluctuations resulted from RNAP^549^ movements relative to the template DNA and not from transient sticking of the DNA to the surface. Similar large intensity fluctuations were not observed before or during the transcript probe signal, indicating that the movements are specific to the post-termination state. Measurements of RNAP^549^ position along the DNA derived from fitting the elongation portion of the record (Supplementary Fig. 2J) revealed that the post-termination fluctuations had the characteristics of a bounded one-dimensional random walk (Fig. 2b, teal), frequently extending over the full ~2 kbp template DNA length (e.g., Supplementary Fig. 2K). In some instances, the intervals of random motion were interspersed with periods of no apparent motion, during which the diffusion coefficient was zero within experimental uncertainty (e.g., Fig. 2a, b purple).

Consistent with post-termination RNAP^549^ molecules switching between a state in which they slide randomly along DNA and a state in which they remain stuck at a fixed position, the distribution of measured diffusion coefficients was bimodal with peaks at ~0 and ~3.5 × 10^4^ bp^2^ s^−1^ (Fig. 2c). Sliding diffusion coefficients of the latter magnitude are below the calculated upper limit for a protein of this size to randomly slide along the DNA helix^30,31^. In a supplementary experiment in which a promoter-ablated mutant template was exposed to core RNAP^549^ in the absence of NTPs and σ^70^ (i.e., conditions in which neither promoter complexes nor elongation complexes should occur), qualitatively similar behavior was observed (Supplementary Fig. 3A and C), indicating that sliding/sticking motion on DNA may be an intrinsic property of core RNAP. In contrast, most σ^70^RNAP holoenzyme molecules exhibited much shorter interactions with DNA under the same conditions and showed few intensity fluctuations indicative of sliding (Supplementary Fig. 3B and C). However, we cannot exclude the possibility that sequence-nonspecifically bound σ^70^RNAP slides but its sliding is not detectable by the methods used here owing to the short duration of most of its DNA associations. Nevertheless, since long-duration sliding/sticking is seen with core RNAP and not with σ^70^RNAP, we speculate that it is a process distinct from any sliding on DNA that may or may not accompany promoter search by holoenzyme^30,32,33^.

### Post-termination complexes can initiate antisense transcripts

**Fig. 3:**
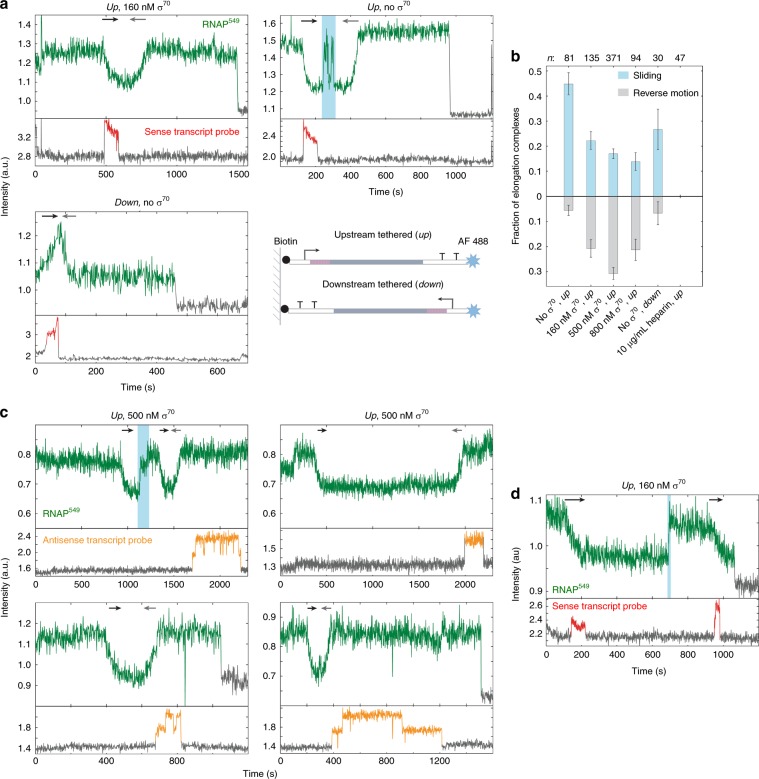
The post-termination RNAP-DNA complex can re-initiate transcription in the antisense direction. **a** Single-molecule emission records, as in Figs. 1c, 2a, for three different DNA spot locations from three separate experiments: one with free σ^70^ in solution and *up* template (upper left), one with no free σ^70^ in solution and *up* template DNA (upper right), and one with no free σ^70^ in solution and *down* template DNA (lower left). Black and gray arrows designate episodes of forward and reverse unidirectional motion corresponding to the directions of sense and antisense transcription, respectively. Teal indicates an interval of RNAP^549^ random sliding. Lower right: Schematics of *up* and *down* templates using the same color scheme as Fig. 1a. **b** Fraction (±s.e.m.) of RNAP^549^ molecules retained at termination (black plus blue populations in Fig. 1d) that slide (teal) or exhibit reverse motion (gray) following termination under different experimental conditions with *up* or *down* templates. **c** Single-molecule emission records as in (**a**) with *up* template DNA and 500 nM free σ^70^ in solution and containing a transcript probe that is the reverse complement of that used in (**a**). **d** Single-molecule emission record as in (**a**) showing post-termination sliding (teal) followed by secondary initiation in the sense direction.

To test whether the sliding RNAP could rebind σ^70^ and initiate a new cycle of transcription, we performed further experiments, in which we introduced σ^70^ free in solution at the time of NTP introduction. The presence of free σ^70^ caused the behavior of most RNAP^549^ molecules retained after termination to change dramatically. Instead of the episodes of fast bi-directional sliding observed in the absence of σ^70^, we often observed slower unidirectional motion of RNAP^549^ in the opposite direction from the initial motion of transcript elongation: RNAP started near the promoter-distal end of the template DNA and moved towards the promoter (Fig. 3a top left, gray arrow). Similar reverse unidirectional motion was also observed in a minority of cases even in the absence of free σ^70^ (e.g., Fig. 3a, top right, gray arrow). In these cases, the reverse motion most often occurred after a brief period of sliding (e.g., teal regions in Fig. 3a and Supplementary Fig. 2B), but it sometimes followed the forward motion with no discernable intervening sliding.

We performed analogous experiments using an otherwise identical template DNA molecule that was tethered to the surface by its downstream instead of its upstream end (Fig. 3a, bottom). Forward followed by reverse motion was seen in the inverted template (e.g., Fig. 3a, bottom right) in the expected directions (i.e., movement toward the surface followed by movement away from the surface). These observations show that reversal of direction is not restricted to the vicinity of the untethered end of the DNA.

The intensity changes corresponding to the forward (black arrows) and reverse (gray arrows) motions on an individual DNA usually exhibited mirror image shapes and similar durations (Fig. 3a and Supplementary Fig. 4A). We hypothesize that the reverse motion reflects synthesis of an antisense transcript. Since core RNAP concentrations in solution are negligible in these experiments^26^, and since we do not observe RNAP dissociation/re-association from DNA, antisense synthesis must be by the same RNAP molecule that had just synthesized and terminated a sense transcript during the forward motion. This hypothesis predicts that a second initiation is required to produce the antisense transcript. Consistent with this prediction, the reverse motions were more frequent, occurring in up to 30% of elongation complexes, when σ^70^ (which is required for initiation) was present free in solution (Fig. 3b). It should be noted that while the template lacks a known promoter for synthesis of an antisense transcript, it does contain an AT-rich sequence at the terminator that might act as a weak σ^70^ promoter. Both sliding and the reverse motions were absent when the polyanion heparin was present. Polyanions can disrupt the stable complexes that form between core RNAP and fully duplex DNA^34,35^. Thus the heparin sensitivity suggests that the RNAP-DNA complex passes through a fully duplex-DNA intermediate (i.e., one with no open transcription bubble) prior to anti-sense initiation.

To check that the antisense transcript was made by the same RNAP molecule that just completed the sense transcript, we took advantage of the fact that even in highly purified *E. coli* RNAP preparations, each individual enzyme molecule has its own characteristic average transcript elongation rate^36–38^. Accordingly, we observed a broad range of characteristic intensity change rates for both sense and antisense transcription events (Supplementary Fig. 4B). However, the rate for a sense transcription event and the subsequent antisense event on the same DNA molecule were usually identical within experimental error, strongly suggesting that both were performed by the same individual RNAP molecule.

As an additional test of the idea that reverse motion is due to antisense transcription, we performed additional single-molecule transcription experiments (e.g., Fig. 3c) using an antisense transcript probe complementary to the sense transcript probe used in Figs. 1, 2, 3a and b. We found that 68% (78/114) of observed RNAP^549^ unidirectional motions towards the promoter were followed by subsequent antisense transcript probe co-localization, compared to just 2% (5/257) co-localization when no unidirectional motion towards the promoter was observed. These observations confirm that reverse motion was due to antisense transcript synthesis. Consistent with the absence of an antisense terminator sequence in the template, the antisense probe fluorescence was often retained for hundreds of seconds after completion of the reverse motion (e.g., Fig. 3c). Eventual disappearance of antisense probe fluorescence could be explained by dissociation of the run-off transcription complex, slow dissociation of probe from transcript, and/or photobleaching.

In the single-molecule experiments we sometimes observed sliding over long distances prior to secondary initiation (Fig. 3a, top right). It is reasonable to ask whether RNAP ever slid back to the end of the DNA with the sense P_R′_ promoter and performed secondary initiation of a *sense* transcript. In rare cases (<2% of retained RNAP^549^ molecules), in the presence of free σ^70^ in solution, we observed retained RNAP^549^ molecules with intensity records indicating a brief period of sliding followed by re-initiation of transcription in the sense direction (Fig. 3d and Supplementary Fig. 4C), suggesting that secondary initiation can occur in either sense or antisense directions relative to primary initiation. Although antisense secondary initiation was preferred over sense in our data, that might be a characteristic of the DNA sequences used rather than an inherent feature of secondary initiation. Thus, free σ^70^ may confer onto DNA-bound core RNAP the capacity to locate and isomerize with promoter sequences after sliding hundreds of basepairs.

**Fig. 4:**
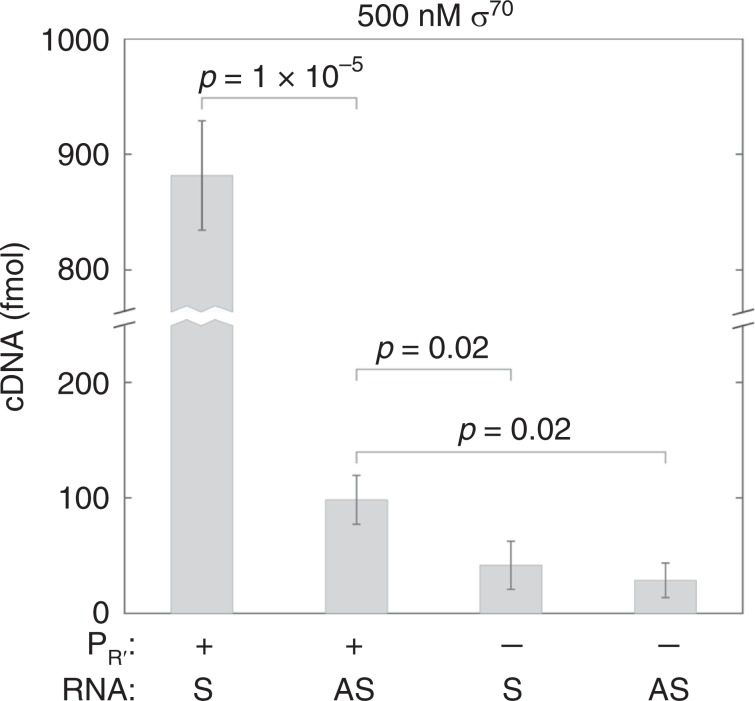
Amounts of both sense and antisense transcripts depend on the sense promoter. Measurements of the amounts of RNAs produced from bulk transcription reactions on templates with an unmodified (Fig. 1a) or a scrambled (“Methods”) λ P_R__′_ promoter. RNAs were reverse transcribed using sense (S) or antisense (AS) specific primers (Supplementary Fig. 5A) and the amounts of cDNA produced were measured by qPCR. Graph shows mean ± s.e.m. of three experiments. All individual measurements from these and additional control samples are shown in Supplementary Fig. 5B.

Antisense transcript was also detected in bulk transcription experiments at 500 nM free σ^70^ by RT-qPCR (Fig. 4 and Supplementary Fig. 5). Consistent with the single-molecule results indicating that the antisense transcript is made by polymerase molecules that have just completed a round of sense transcription, the bulk experiments showed that ablation of the promoter for the sense transcript reduced the concentrations of both sense and antisense transcripts (Fig. 4). In this experiment, the ratio of antisense to sense transcript, 11 ± 2%, was somewhat lower than seen in the single-molecule experiments (31 ± 2% from 500 nM σ^70^ data in Fig. 3b). This difference might result from transcriptional interference^20^ caused by the multiple rounds of initiation possible in the RT-qPCR experiment; the design of the single-molecule experiment allowed only a single round. The RT-qPCR results were obtained with wild-type RNAP, confirming that the antisense transcript is not an artifact of the SNAP tagged and dye-labeled polymerase construct used in the single-molecule experiments. Taken together, the single molecule and bulk experiments show that antisense transcript synthesis on this template in vitro results from secondary initiation by RNAP molecules that first associated with the DNA through prior initiation at the sense promoter.

### Secondary initiation of antisense transcripts in vivo

**Fig. 5:**
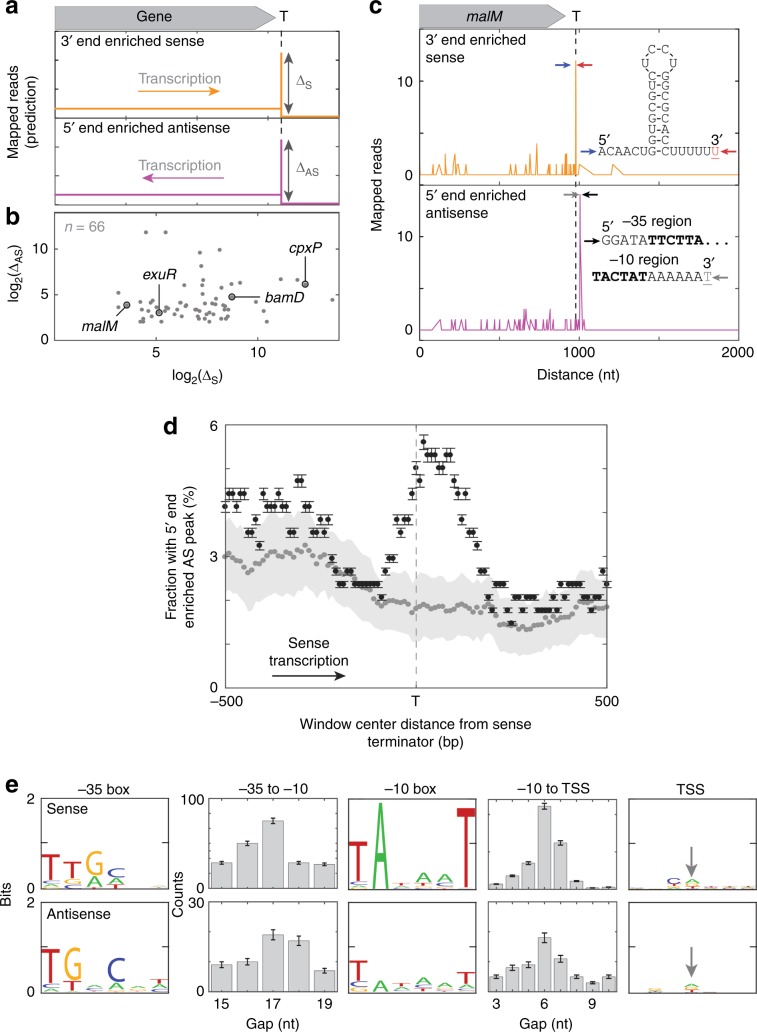
Secondary initiation of antisense transcripts in vivo. **a** Predicted Rend-seq signature of antisense secondary initiation at an intrinsic terminator. Idealized plot shows a genomic region near a terminator (T). Termination is indicated by a peak in 3′ end enriched sense reads (orange). The secondary initiation hypothesis predicts nearby 5′ end enriched antisense reads (magenta), suggestive of antisense secondary initiation. Δ_*S*_ and Δ_*AS*_ are the relative amounts of sense termination and antisense initiation at particular genomic positions as estimated by the peak heights. **b** Peak heights from 66 (of 339 total) terminators detected in *E. coli* that show a substantial Δ_AS_ peak within 500 bp of the terminator Δ_S_ peak. Labels mark the genes shown in (**c**) and in Supplementary Fig. 6A. **c** Example of the phenomenon predicted in (**a**) observed in Rend-seq data from ref. ^39^ near the terminator of the *E. coli malM* gene. Shown are the factor-independent terminator RNA sequence with the peak of sense termination at the underlined red nucleotide, and the promoter-like non-template strand DNA sequence with the peak of antisense initiation at the underlined gray nucleotide. Arrows mark the positions of the displayed sequences in the Rend-seq data. **d** Antisense initiation peak frequency correlates with positions of sense terminators in the *E. coli* genome. Pooled data from 339 terminators between genes transcribed in the same direction (see *Methods*). Plot shows the fraction (±s.e.m.) of 200 nt-wide windows centered at the indicated distance upstream or downstream from the terminators that exhibit a peak of antisense initiation with *z*-score > 12 (black). Also shown is the mean ± s.d. of negative controls (gray) in which the same analysis was repeated 100 times each using 339 randomly selected locations in the *E. coli* genome that lack apparent terminators. These locations were restricted to those >700 nt from an annotated terminator and were on the sense strand of genomic regions containing at least three consecutive genes in the same orientation. In 100% of these 100 control replicates, the fraction at the terminator location with a 5′ end AS peak was <3.9%, indicating that the difference between experimental data and controls was significant (*p* < 0.01). In this analysis, we used a smaller window size than in (**b**) to improve spatial resolution and a very stringent peak height criterion, *z* > 12. This leads to detection of only the strongest peaks and shows that these strong antisense peaks are preferentially found in a region ± 200 nt from sense terminators. **e** Sequence characteristics (illustrated as in ref. ^62^) for *n* = 250 strong sense promoters (top) and for the *n* = 66 terminator-proximal antisense initiation sites shown in (**b**) (bottom), all detected by Rend-seq^39^. Logos show the sequence motifs for the −35 box, −10 box, and transcription start site (TSS, arrow); histograms display the distributions of spacings between these elements. When the size of the −10 box was expanded from six to 8, 9, or 10 bp, there was no strong evidence for extended −10 sequences.

**Fig. 6:**
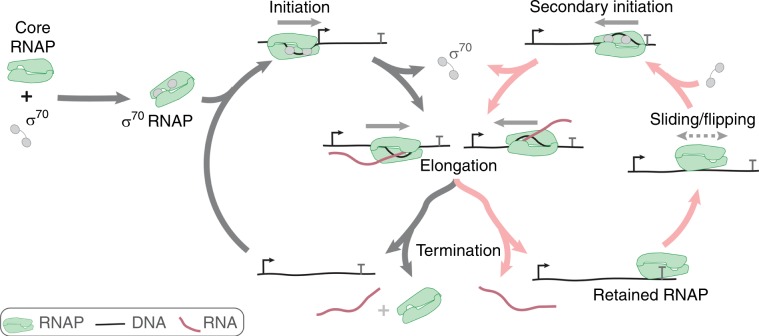
Proposed expanded bacterial transcription mechanism. RNAP retention after termination leads to an expanded pathway for transcript production, consisting of linked canonical (gray) and alternative (red) cycles. See text. Antisense promoter-like and terminator sequences are not shown.

The forgoing experiments demonstrate that secondary initiation occurs in vitro with purified RNAP and on a particular template DNA sequence but leave open the question of whether this phenomenon also occurs in living cells and on other template sequences. To investigate this, we used data from end-enriched RNA sequencing experiments that map the genomic positions of RNA 5′ and 3′ ends (Rend-seq^39^). The secondary initiation hypothesis suggests that intrinsic terminators will be associated with nearby antisense initiation (Fig. 5a). In *E. coli*, active intrinsic termination sites confirmed by Rend-seq data^39^ were (in ~20% of cases) accompanied by highly significant levels (>12 standard deviations above the mean; see the “Methods” section) of a nearby (within 500 bp) RNA 5′ end mapping to the opposite strand, indicative of antisense initiation (Fig. 5b–d). These antisense initiation sites were significantly more frequent near terminators than farther away from them, indicating that the positions of sense terminators and antisense initiation were correlated (Supplementary Fig. 6A). Similar proximity was seen in data from *B. subtilis* (Supplementary Fig. 6B, C, and D), showing that it is a feature common to datasets collected from divergent species. Furthermore, the height of the antisense initiation peak was often increased in data from a *B. subtilis* strain with deletion of the Rho termination factor gene, consistent with prior observations that steady-state levels of antisense transcripts are greatly increased by Rho mutation or inhibition^39–42^. Both species display a −10 box motif at sites of antisense initiation that is similar to the −10 box motif at sense initiation sites^43^, confirming that antisense initiation occurs at promoter-like sequences (Fig. 5e and Supplementary Fig. 6E; compare ref. ^44^). In contrast, the antisense initiation peaks have −35 boxes different from those of sense initiation peaks when analyzed with the same algorithm^45^; see the “Methods” section. The −35 box is usually the most important sequence determinant in initial recruitment of RNAP to the promoter. The different sense and antisense −35 box sequences reported here may indicate that different sequences are optimal for different recruitment processes (e.g., binding of RNAP from solution for sense initiation vs. RNAP already bound nearby on DNA for antisense secondary initiation). Taken together, these RNA sequencing analyses show that antisense initiation occurs preferentially near some terminators, and occurs at discrete promoter-like sequences with characteristics distinct from those of sense promoters. Such promoter-like sequences near terminators might be selected for (or, in other contexts, against) during genomic evolution. Sequencing does not follow individual RNAPs and thus cannot establish that sense and antisense RNAs are made sequentially by the same polymerase molecule. However, the data show antisense production that is consistent with the mechanism of secondary initiation (Fig. 6) deduced from our experiments in vitro.

## Discussion

Taken together, our results suggest an expanded version of the bacterial transcription pathway (Fig. 6), in which core RNAP retention on DNA after intrinsic termination can lead to synthesis of antisense (and possibly of additional sense) transcripts. In the canonical transcription cycle (Fig. 6, gray arrows) holoenzyme formed by association of a sigma protein with core RNAP initiates at a sense transcription promoter and elongates a transcript. Sigma is released from most elongation complexes. The transcript and polymerase dissociate from template DNA upon reaching a sense transcript terminator (T) sequence. In the alternative cycle (Fig. 6, red arrows), the termination process is different: core RNAP is retained on DNA after RNA is released at the terminator. This retained polymerase, which we assume is making only sequence non-specific interactions with the backbone of a fully base paired DNA, undergoes diffusional sliding along DNA. We further assume that the sliding RNAP molecule, like other sequence non-specific protein DNA complexes (see below), can occasionally flip its orientation on the DNA through transient dissociation and rapid rebinding. While in this sliding state, RNAP may bind a sigma factor, encounter a promoter-like sequence with orientation matching that of the polymerase, open a bubble in the DNA, and initiate a new transcript (secondary initiation) in a direction opposite to or the same as the direction of transcription before termination. Opposite-direction secondary initiation produces an antisense transcript. Each elongation complex is assumed to be capable of stochastically selecting either the canonical or the alternative cycle at the time of termination. Many of our findings of RNAP initiation after termination are consistent with independent observations reported by Kang et al.^46^, adding credence to the phenomena.

The nascent transcript dissociates rapidly (in ~0.5 s) from RNAP when an EC reaches an intrinsic terminator^8^. However, the rate of DNA release from RNAP at terminators is controversial; early studies produced indirect evidence for both rapid (seconds) and slow (minutes) release (ref. ^5^ and refs. cited therein). More recent studies show that at intrinsic terminators RNA release from RNAP occurs first and that an RNAP conformational change precedes subsequent DNA release^6^. In the single-molecule experiments, we directly measured the time between RNA release and DNA release and found that DNA release takes on average >10 min in the absence of free σ^70^. This is consistent with earlier single-molecule observations^8^ but superficially contradicts later work^7^ which saw associations lasting only a fraction of a second. However, in those experiments the RNAP-DNA complexes were held under >3 pN tension in an optical trap, a force predicted (see the “Methods” section) to move the sliding complexes we observe to the end of the DNA in 0.05 s. The optical trap data are consistent with our observations if one postulates that in those experiments the RNAP rapidly dissociated once it was pulled to the DNA end.

Early work characterizing RNAP-DNA interactions showed that core RNAP binds non-promoter DNA substantially more tightly than does σ^70^RNAP^5,34,35^. However, there has been no known role for core RNAP-DNA interactions in the absence of the transcription bubble and nascent RNA present in an EC. Here, we validate previous proposals^5,47^ by showing that a core RNAP-DNA complex is a transcription cycle intermediate that is often produced upon transcript release at one or both of the intrinsic terminators used in our experiments. These post-termination complexes are kinetically stable, and they can exhibit long-range sliding along DNA. Evidence that most or all of the sliding complexes contain core RNAP rather than holoenzyme includes: (1) the fraction of complexes that we see slide post-termination (Fig. 3b) is much larger than the fraction that retain σ^70^ (21%; see ref. ^26^); (2) adding σ^70^ to the solution suppresses post-termination sliding (Fig. 3b); and (3) incubating core RNAP with promoterless DNA can produce a population of similarly long-lived sliding complexes (Supplementary Fig. 3). The behaviors observed in the presence of free σ^70^ further suggest that after termination, the sliding core RNAP-DNA complexes can bind σ^70^ and re-initiate transcription. The rare (<5%; Fig. 3b) post-termination complexes that exhibit secondary initiation in the absence of added σ^70^ presumably are complexes that retained σ^70^ during primary transcription^26^. We speculate that sliding-mediated secondary initiation represents a previously unknown biological function of the kinetically stable core RNAP-DNA interaction.

Our results suggest that after one round of transcription, RNAP can initiate a second round in the opposite direction without intervening dissociation and diffusion of the enzyme away from the DNA. This “flipping” presumably requires RNAP to rotate by 180 degrees about an axis normal to the DNA helix. Although flipping has not previously been reported for RNAPs, it has multiple precedents in other enzymes that slide on or move processively along nucleic acids (ref. ^48^ and references cited therein; ref. ^49^). In those enzymes, flipping is presumed to occur via undetectable brief dissociation limited to the microsecond/nanometer scale followed by rapid rebinding of the protein to the DNA^48,50^. It is conceivable that in bacterial RNAPs, an α subunit C-terminal domain^51^ could increase the efficiency of flipping by flexibly tethering RNAP to DNA while it rotates.

Although secondary initiation has not previously been reported for bacterial RNAP, there is evidence that the same molecule of eukaryotic RNA polymerase III can re-initiate a second round of sense transcription at the same promoter after termination of the first round^52,53^. While this re-initiation has been proposed to occur by a looping or “handing back” mechanism mediated by transcription factors, our results with bacterial RNAP suggest sliding as a possible alternative mechanism.

Antisense transcription is known to act through transcription interference and other processes to regulate specific genes in bacteria^10,17^, and terminator/antisense promoter modules have been shown in synthetic genetic constructs to exert a general suppressive effect on transcript production from the upstream sense gene^23^. Antisense transcription, including transcripts that initiate downstream of sense terminators, is pervasive in bacteria, but the mechanisms that give rise to it are not well understood and antisense promoter sequences are not well conserved^14^. The retention after termination/sliding/flipping mechanism described here is noteworthy because antisense transcript production immediately follows and is coupled to the production of a sense transcript from the same gene by the same RNAP molecule. Thus, initiation at the sense promoter can directly produce an antisense transcript to down-regulate sense gene expression. This mechanism could provide a fast-acting negative feedback that suppresses spurious expression in bacteria without the time required for translation, serving a regulatory role similar to that reported in regulation of eukaryotic transcription^18^. In addition, our observations raise the possibility that the presence of a sense promoter(s) near an intrinsic terminator could cause RNAP retained after intrinsic termination to do secondary initiation in the sense direction from the nearby promoters. This could serve as a gene coupling mechanism in which transcription from an operon could serve to activate adjacent promoters, leading to local regions of enhanced transcription in the bacterial genome. Further study will be required to elucidate the gene-specific roles of these molecular behaviors in living cells.

## Methods

### Template DNA and oligonucleotides

Circular transcription templates were the plasmids pCDW114 (GenBank accession no. KT326913) and pCDW116. Plasmid pCDW116 has the same sequence as pCDW114 but the P_R’_ –35-box TATTGACT in pCDW114 was mutated to CAGGCGCT. Linear transcription templates (*up* and *down* DNA^488^, Figs. 1a and 3a) were synthesized by PCR from plasmids pCDW114 and pCDW116 using the primers p397 and p447 (Supplementary Table 1). The template lacking a P_R__′_ promoter sequence (Fig. 4; Supplementary Figs. 3 and 5) was synthesized in the same way using plasmid pCDW116. The 20nt Cy-5-labeled sense and antisense transcription probes were 5′-GTG TGT GGT CTG TGG TGT CT/3Cy5Sp/-3′ and 5′-AGA CAC CAC AGA CCA CAC AC/3Cy5Sp/-3′, respectively (IDT, Coralville, IA).

### Proteins

*E. coli* core RNAP (αββ′ω) with a SNAP tag on the c-terminus of β´ (RNAP-SNAP) and wild-type σ^70^ protein were expressed and purified^54^. RNAP-SNAP was labeled with the DY-549 dye, yielding RNAP^549^, as follows: 20 μL of 15 μM RNAP-SNAP was dialyzed into 3 L of labeling buffer (10 mM Tris-HCl, pH 8.0, 40 mM KCl, 5 mM MgCl_2_, 20 μM ZnCl_2_, and 1 mM dithiothreitol (DTT)) at 4 °C for 4 h. The resulting product (typically 50–100 μL of 5–20 μM of protein) was mixed with an equimolar amount of SNAP-Surface 549 (New England Biolabs; 1 mM in DMSO) and incubated at room temperature for 30 min, then mixed with an equal volume of labeling buffer supplemented with 60% glycerol to yield RNAP^549^ in reconstitution buffer (10 mM Tris-HCl, pH 8.0, 30% glycerol, 0.1 mM EDTA, 100 mM NaCl, 20 mM KCl, 20 μM ZnCl_2_, 3 mM MgCl_2_, and 0.6 mM DTT). The preparation was flash frozen in liquid N_2_ and stored at -80 °C.

σ^70^RNAP^549^ holoenzyme was prepared by incubating equimolar σ^70^ and RNAP^549^ in reconstitution buffer at 37 ˚C for 10 min and then stored at −20 °C for up to 3 h before use.

### Single molecule transcription experiments

Single-molecule total internal reflection fluorescence microscopy was performed at excitation wavelengths 488, 532 and 633 nm, for observation of DNA^488^ template, RNAP^549^ and Cy5-transcript probe, respectively^27^; focus was automatically maintained^55^. Transcription reactions were conducted as described^26^. Briefly, single-molecule observations were performed in glass flow chambers (volume ~20 µL) passivated with succinimidyl (NHS) polyethylene glycol (PEG) and NHS-PEG-biotin (Laysan Bio Inc.; Arab, AL)^27^. Streptavidin (#21125; Life Technologies; Grand Island, NY) was introduced at 220 nM in wash buffer (50 mM Tris acetate, 100 mM potassium acetate, 8 mM magnesium acetate, 27 mM ammonium acetate, 0.1 mg mL^−1^ bovine serum albumin (BSA) (#126615 EMB Chemicals; La Jolla, CA), pH 8.0), incubated 45 s, and washed out (this and all subsequent wash out steps used two flushes each of four chamber volumes of wash buffer). The chamber was then incubated with 50 pM AF488-DNA in wash buffer for ~2 min and washed out. Next, locations of surface-tethered AF488-DNA molecules were recorded by acquiring four 1 s images with 488 nm excitation at a power of 350 µW incident to the objective lens^55^.

For transcription reactions σ^70^RNAP^549^ holoenzyme was introduced into the chamber at 1 nM in transcription buffer (wash buffer supplemented with 3.5% w/v PEG 8,000 (#81268; Sigma-Aldrich; St. Louis, MO), 1 mg mL^−1^ BSA, and an O_2_-scavenging system^56^, incubated for ~10 min, and washed out. Finally, we started image acquisition (iterations of thirty 1 s exposures to simultaneous 532 and 633 nm excitation, each at 200 µW, followed by four 1 s exposures to 350 µW 488 nm excitation) and initiated transcription by introducing transcription buffer supplemented with 500 µM each of ATP, CTP, GTP and UTP, and 10 nM Cy5-probe.

Image analysis was done using custom software and algorithms for automatic spot detection, spatial drift correction and co-localization^57^.

### Bulk transcription experiments

Open-promoter complexes were formed by combining 8.8 nM unlabeled σ^70^RNAP-SNAP holoenzyme with 8 nM of DNA template in 50 µL of transcription buffer supplemented with 660 nM σ^70^ and incubated for 5 min. Transcription was then initiated by the introduction of 500 μM each of ATP, CTP, GTP, and UTP. The reaction was allowed to proceed for 40 min at room temperature; at that time total RNA was purified using RNeasy mini Kit (Qiagen; Cat No. 74104) column and protocol including on-column RNase-free DNase digestion (Qiagen; Cat No. 79254) and eluted into 30 μL RNase free water.

### RT-qPCR

First strand complementary DNA (cDNA) was synthesized in a 25 µL reaction containing 12.5 µL sample RNA, 2 pmol strand-specific cDNA primer (Supplementary Fig. 5A) and 200 units SuperScript IV reverse transcriptase (ThermoFisher; Cat No.18090010) in RT buffer (50 mM Tris-HCl, pH 8.3, 50 mM KCl, 3 mM MgCl_2_, 10 mM DTT, and 1 mM each dATP, dCTP, dGTP, and dTTP) and incubated according to the SuperScript IV reverse transcriptase protocol. cDNA product was diluted 1:2 into TE buffer (10 mM Tris-HCl pH 8.0, 0.1 mM EDTA). qPCR was conducted using qPCR primers chosen to amplify the cDNA (Supplementary Fig. 5A) in 20 μL reactions containing 4 µL diluted cDNA, 0.5 μM primers, 0.2 µL Herculase II Fusion DNA Polymerase (Agilent Technologies; Cat No 600675), and Sybr Green (ThermoFisher) at the manufacturer’s recommended concentration. cDNA synthesis reactions were performed on three different days; subsequent to each cDNA reaction qPCR was performed in triplicate on each sample. On each day, sense and antisense standard curves were measured from nine qPCR reactions containing known amounts of target sequence double stranded DNA (6 × (10^2^, 10^3^, 10^4^, 10^5^, 10^6^, 10^7^, 10^8^, 10^9^ or 10^10^)) molecules. Sense or antisense cDNA copy number in each qPCR reaction was calculated using parameters derived from fitting the corresponding standard curve. Mean qPCR amplification efficiency was 104 ± 4%.

### Characteristic lifetime of RNAP^549^

To measure the characteristic lifetime of retained RNAP^549^, we jointly fit to an exponential probability distribution the measured lifetimes of retained RNAP^549^ that terminated by disappearance of the fluorescent spot and those that were censored by halting image acquisition using the maximum likelihood algorithm, yielding the reciprocal time constant *k*_obs_^26^. The dissociation rate of retained RNAP^549^, *k*_RNAP_, was computed by *k*_RNAP_ = *k*_obs_−*k*_PB_ where *k*_PB_ is the rate of RNAP^549^ photobleaching (Supplementary Fig. 1B) and the characteristic RNAP lifetime was calculated as 1/*k*_RNAP_. Errors were calculated by bootstrapping^57^ and error propagation.

### Measurement of RNAP^549^ position on template

We used location-specific calibration curves at the position of each DNA molecule to convert measured RNAP^549^ fluorescence intensity to position along the DNA contour. To define the calibration curve, we first fit the RNAP^549^ fluorescence record during the period of steady-state elongation (Supplementary Fig 2J, black arrow) to the expression

1 $${\boldsymbol{I}}\left( {\boldsymbol{t}} \right)\, = \, {\boldsymbol{I}}_{\mathbf{P}}{\boldsymbol{e}}^{-{\mathbf{\lambda}}{\boldsymbol{t}}}\, + \, {\boldsymbol{I}}_{{\mathbf{{mn}}}}$$

where *I*_P_ and *I*_mn_ are the fluorescence intensity of the promoter-bound RNAP^549^ and the mean magnitude of the background fluorescence as depicted in Supplementary Fig. 2J, and the fit parameter *λ* is the decay constant (Supplementary Fig. 2J, blue curve). We assumed the rate of elongation was constant^36–38^, yielding the relationship

2 $${\boldsymbol{z}}\left( {\boldsymbol{t}} \right) = {\boldsymbol{r}}_{{\mathbf{RNAP}}}{\boldsymbol{t}} + {\boldsymbol{z}}_{\mathbf{P}}$$

where $z(t)$ is the position of the polymerase along the contour of the DNA during elongation, *r*_RNAP_ is the rate of RNAP elongation, and *z*_p_ is the position of the promoter along the DNA contour. Taking the time of probe release as the time of termination, we measured the fluorescence intensity at termination, *I*(*t*_T_) = *I*_T_ (Supplementary Fig. 2J), and used it to compute *r*_RNAP_ by combining Eqs. 1 and 2 and using the known position of the terminator along the DNA contour *z*_T_:

3 $${\boldsymbol{r}}_{{\mathbf{RNAP}}} = \frac{{{\boldsymbol{z}}_{\mathbf{T}} - {\boldsymbol{z}}_{\mathbf{P}}}}{{{\boldsymbol{1/}}{\mathbf{\lambda }}\;{\boldsymbol{ln}}\frac{{{\boldsymbol{I}}_{\mathbf{P}}}}{{{\boldsymbol{I}}_{\mathbf{T}} - {\boldsymbol{I}}_{{\mathbf{mn}}}}}}}$$

Finally, combining Eqs. 1 and 2 yields an expression relating the time-dependent position of the polymerase on the DNA contour to the measured time-dependent fluorescence intensity *I*(*t*) after termination

4 $${\boldsymbol{z}}\left( {\boldsymbol{t}} \right) = {\boldsymbol{r}}_{{\mathbf{RNAP}}}\frac{1}{{\mathbf{\lambda }}}{\boldsymbol{ln}}\frac{{{\boldsymbol{I}}_{\mathbf{p}}}}{{{\boldsymbol{I}}\left( {\boldsymbol{t}} \right) - {\boldsymbol{I}}_{{\mathbf{mn}}}}} + {\boldsymbol{z}}_{\mathbf{P}}$$

in terms of known and measured parameters. An example record of *z*(*t*) is shown in Supplementary Fig. 2K.

### Identifying sliding and antisense transcription behavior

To measure the fractions of retained RNAP^549^ molecules that exhibited post-termination sliding or antisense transcription (Fig. 3b), we analyzed all RNAP^549^ fluorescence emission records that displayed sense transcript elongation as judged by fluorescence intensity changes. Sliding was scored if any 50 s time window following the RNAP^549^ elongation signature contained a measured diffusion coefficient, *D*, of 2.2 × 10^4^ bp^2^ s^−1^ or greater. This *D* value corresponds to the local minima of the saddle point in Fig. 2c. Antisense transcription was scored if a region of the RNAP^549^ intensity record after sense transcript elongation completed exhibited a visible antisense elongation profile that when fit had an exponential time constant between 0.002 and 0.04 s^–1^.

### Estimate of post-termination RNAP drift velocity under force

Previous work^7^ employed an optical trapping assay featuring bead-tethered RNAP undergoing steady-state elongation on surface-tethered template DNA, with tension (as small as 3 pN) imposed between the two by the trap. Upon RNA reaching the position of an intrinsic terminator on the DNA, dissociation of RNAP from DNA was detected as loss of the mechanical linkage between bead and surface. These data were interpreted as sub-second dissociation of RNAP from DNA after intrinsic termination. Here we claim that our observation of a long-lived (hundreds of seconds), DNA-bound sliding RNAP state following intrinsic termination is fully consistent with the sub-second dissociation seen under applied force.

The Einstein–Smoluchowski equation relates the one-dimensional diffusion constant of a particle, *D*, to the drift velocity, *v*_d_, under an external force, *F*:

5 $${\boldsymbol{D}} = {\boldsymbol{k}}_{\boldsymbol{B}}{\boldsymbol{T}}\frac{{{\boldsymbol{v}}_{\mathbf{d}}}}{{\boldsymbol{F}}}$$

where *k*_*B*_ is Boltzmann’s constant and *T* is temperature. Solving Eq. 5 for *v*_d_ and evaluating the expression using our measured diffusion constant of RNAP on DNA, *D* = 4 × 10^4^ bp^2^ s^−1^ = 4 × 10^3^ nm^2^ s^−1^, the minimum external force imposed on RNAP relative to DNA in ref. ^7^, *F* = 3 pN, and *T* = 300 K, yields the drift velocity of the post-termination RNAP in the sliding state under the external force imposed by the optical trap: *v*_d_ = 3 × 10^3^ nm s^−1^. At this drift velocity, RNAP in the optical trap assay will be pulled along the DNA from the position of the terminator to where it could slide off of the blunt end (~150 nm) in ~0.05 s, consistent with the rapid dissociation observed in those experiments.

### Intrinsic termination and antisense transcription in vivo

To establish a reference set of terminators for analysis, we used sets of 630 *E. coli* and 1486 *B. subtilis* terminators with terminator function in vivo established by experimental data on wild-type strains^39^. To ensure that the identification of 5′ ends was not affected by peak shadows near the ends of convergent genes^39^, we restricted our analysis to a subset of *n* *=* 339 (*E. coli*) or 726 (*B. subtilis*) terminators for which the nearest upstream and downstream genes were annotated in the reference genome NC_000913.2 (*E. coli*) or NC_000964.3 (*B. subtilis*) to be in the same orientation as the terminator. To quantify sense termination, we first defined *k*_max_ as the peak number of 3′ end-enriched reads mapped to the same strand as the terminator in a 10 bp region around each terminator. The magnitude of sense termination was taken to be Δ_S_ = log_2_(*k*_max_) (Fig. 5b and Supplementary Fig. 6B). To estimate the effect of the terminators on antisense transcript production, we first defined *k*_max2_ as the peak count of 5′ end-enriched reads mapped to the opposite strand in a ±500 bp region around each terminator. The magnitude of antisense initiation was taken as Δ_AS_ = log_2_(*k*_max2_). Antisense initiation peaks were identified by *z*-score transformation^39^; a threshold of *z*-score > 12 was used to select strong peaks (*n* = 66 *E. coli* or *n* = 117 *B. subtilis* Δ*rho* terminators met this criterion).

### Promoter sequence characteristics

To determine the sequence characteristics of the transcription start site (TSS) (Fig. 5e and Supplementary Fig. 6E), at each antisense initiation peak, we first measured the information content of the nucleic acid sequence^43^ in a ±3 bp window centered on each peak. To determine the sequence characteristics of the −10 box, as well as the distribution of gap lengths between the TSS and −10 box, we used BIPAD, a web server for modeling bipartite sequence elements with variable spacing^45,58^. After substituting 7A nucleotides for positions +1 through +7 (relative to the TSS at +1), we fit positions −20 to +7 (BIPAD parameters: gap range, 3–10 bp; widths of sequence elements, 6 bp and 7 bp; 1000 runs). To determine the sequence characteristics of the −35 box, as well as the distribution of gaps between the −10 box and −35 box, positions −43 to −3 were fit (BIPAD parameters: gap range, 15-19 bp; two sequence-element search; widths, 6 bp and 6 bp; 1000 runs). For comparison, the same analysis was used on sets of *E. coli* and *B. subtilis* sense initiation peaks detected in Rend-seq data. These sense initiation peaks were identified by peak *z*-score > 12 (ref. ^39^) in wild-type cells.

### Reporting summary

Further information on research design is available in the Nature Research Reporting Summary linked to this article.

## Supplementary information

Supplementary Information

Peer Review File

Reporting Summary

## Data Availability

Single-molecule data were collected on a custom built total internal reflection fluorescence microscope operated using open-source software available at https://github.com/gelles-brandeis/Glimpse. Single-molecule data were analyzed by the Matlab program imscroll available at https://github.com/gelles-brandeis/CoSMoS_Analysis.

## Data availability

A reporting summary for this Article is available as a Supplementary Information file. Source data for Figs. 1b, c, d; 2a, b, c; 3a, b, d; 4; 5b; and Supplementary Figs. 1A, B, C, D, E, F; 2; 3 A, B, C; 4A, B, C; and 5 are archived at https://zenodo.org as 10.5281/zenodo.3476736. In this archive, single-molecule source data are provided as “intervals” files that can be read and manipulated by the Matlab program imscroll, which is publicly available: https://github.com/gelles-brandeis/CoSMoS_Analysis. The archive includes an index file that identifies which data files correspond to which curves in which figure panels. All data is available from the corresponding author upon reasonable request.
