# Supplementary material for: Alternative transcription cycle for bacterial RNA polymerase
Source: Nat Commun. 2020 Jan 23;11:448. doi: 10.1038/s41467-019-14208-9 (PMC6978322; doi:10.1038/s41467-019-14208-9)
Supplement: Supplementary file 1 — Supplementary Information [file 41467_2019_14208_MOESM1_ESM.pdf]

Supplementary Information for Harden *et al.*

Alternative transcription cycle for bacterial RNA polymerase

Supplementary Figures 1-6

Supplementary Table 1

Supplementary References

SUPPLEMENTARY FIGURES

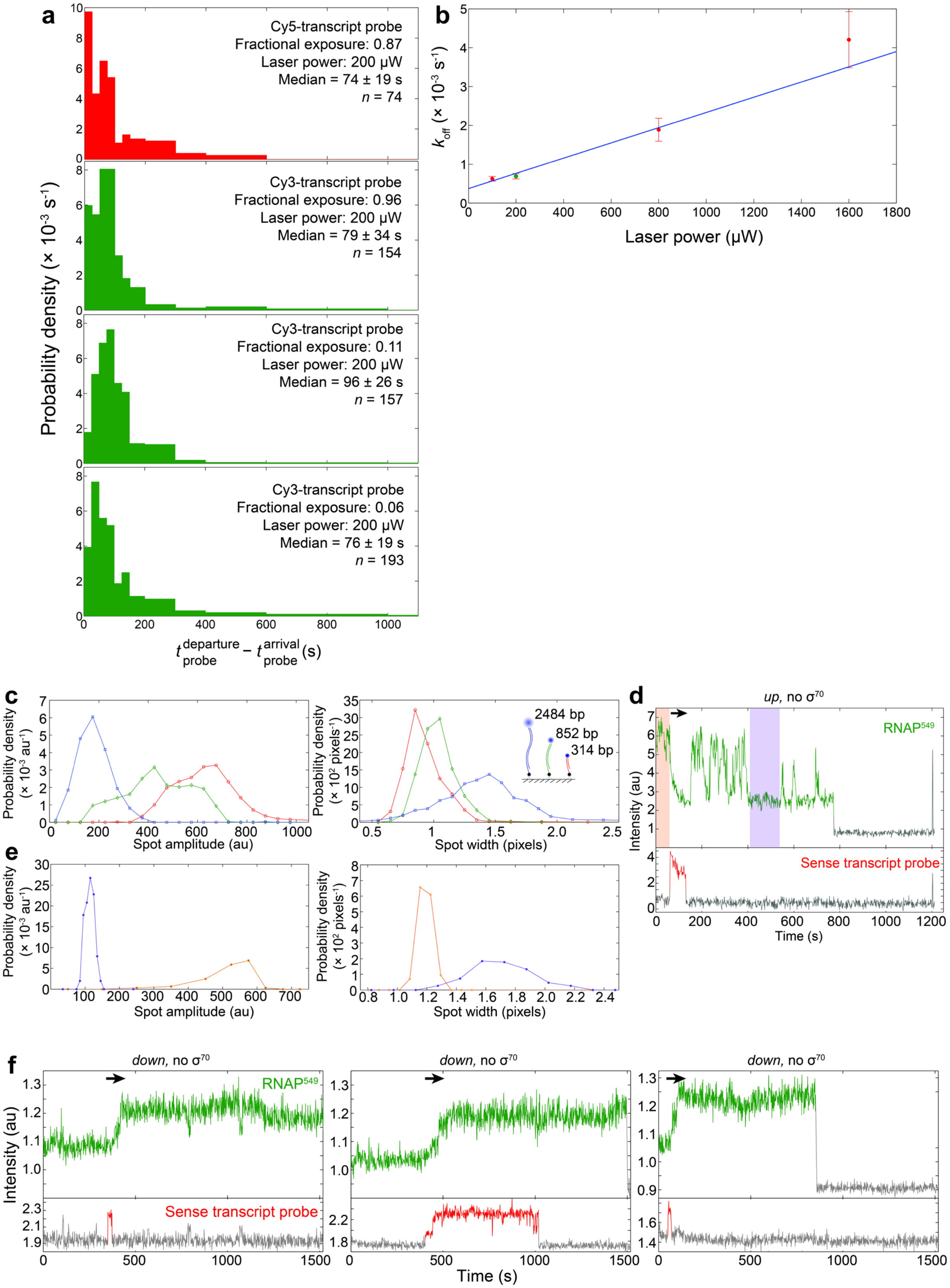

Supplementary Figure 1. Validation of single-molecule experiments to observe pre- and post-termination RNAP-DNA complexes. Effects of laser exposure on single-molecule photobleaching lifetimes of fluorescently tagged species. (**A**) Normalized histograms of transcript probe lifetimes with either a Cy5 (red) or Cy3 (green) labels on the probe, measured at the indicated fractional exposures (i.e., the fraction of the time the sample is

exposed to the excitation laser). A two-sided Kolmogorov-Smirnov test between each distribution ($p = 0.95$) failed to reject the null hypothesis that each of the distributions were selected from the same parent distribution. The red histogram corresponds to the conditions used in this study; the green histograms are taken from ref. [1]. The similarity of the green lifetime distributions over a 16-fold range of fractional exposure established that the measured Cy3 probe lifetimes are not limited by photobleaching. The similarity of these distributions to that of the Cy5-probe lifetimes suggests that the latter are also not significantly limited by photobleaching[1]. (**B**) Measurements of the first-order fluorescent spot disappearance rate constant k_{off} of RNAP[549] in open-promoter complexes with template DNA at varying excitation laser powers. The point at 200 μW (green) corresponds to the power used in the experiments reported in the study. Linear fit (line) yielded intercept $(3.7 \pm 0.4) \times 10^{-4}$ s^{-1} (the open complex dissociation rate after photobleaching correction) and slope $(2.0 \pm 0.8) \times 10^{-6}$ s^{-1} μW^{-1}, corresponding to a photobleaching rate at 200 μW of $k_{\text{PB}} = (7 \pm 2) \times 10^{-4}$ s^{-1} or photobleaching lifetime $\tau = 1300 \pm 300$ s, significantly more than the median lifetime of the transcript probe (75 ± 18 s). (**C, D, E**) Tethered fluorophore motion reports movement of RNAP along DNA. **(C)** Control experiment with three different lengths of dye-labeled DNA confirms a previous report[2] that florescence spot intensity and width change systematically with DNA tether length due to tethered fluorophore motion (TFM). Three DNA species of lengths 2484 bp (blue), 852 bp (green) and 314 bp (red), each modified with an AF488 dye at one end and biotin at the other, were sequentially introduced into a streptavidin-derivatized flow chamber. After each introduction, the locations of molecules of that length were recorded for subsequent analysis. A recording of a single field of view with all three molecular species present was then analyzed by fitting each fluorescent spot with a two-dimensional Gaussian[3]. Histograms show the resulting spot amplitudes (left) and width (i.e., the standard deviation of the Gaussian; right) classified by DNA type. Analysis was conducted on 10 images (each 1s exposure time) containing 70 (blue), 53 (green) and 102 (red) DNA molecules, producing 700, 530, and 1020 measurements, respectively in the three distributions. (**D**) The same experimental record shown in Figure 2A. Shading indicates two time intervals chosen to be prior to transcript elongation (salmon) and after termination (purple) as judged by the transcript probe signal. (**E**) Histograms of spot amplitude (left) and width (right) demonstrating detection of RNAP[549] movement on DNA during the single-molecule transcription event depicted in (D). Spot images used to produce the salmon ($n = 61$) and purple ($n = 101$) histograms were drawn from the indicated time intervals in (D). Before elongation, RNAP[549] is located closer to the surface than it is during the selected interval after termination, as demonstrated by the lower amplitude and greater width in the purple distributions relative to the salmon. (**F**) Example records illustrating RNAP transcribing a template DNA that is inverted relative to the template orientation used in Figure 1. Records selected from an experiment with *down* (*Methods*) template DNA showing RNAP[549] and Cy5-transcript probe emission co-localized with 3 different DNA spots, plotted as in Figure 1C. Gray color marks intervals during which no fluorescent spot was detected. Arrows mark intervals of transcript elongation. This template is attached to the surface in the opposite orientation from that in Figure 1A, and the fluorescence intensity increases, rather than decreases, during transcript elongation as expected.

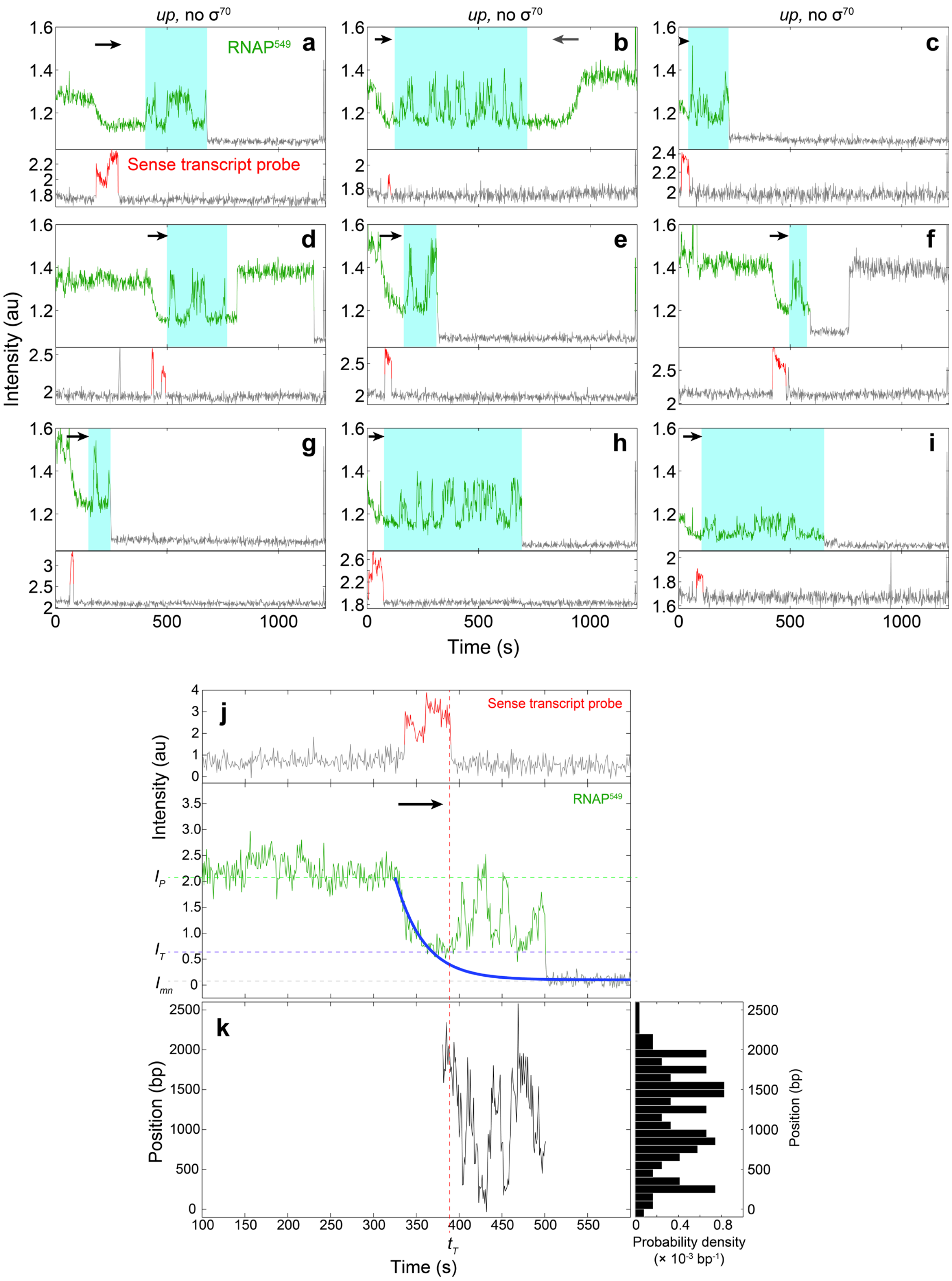

Supplementary Figure 2. Example records illustrating RNAP sliding on DNA after termination and measuring retained RNAP^{549} position on template DNA. (**A-I**) Records selected from the experiment in Figures 1 and 2 showing RNAP^{549} and Cy5-transcript probe emission co-localized with nine different DNA spots. Gray color marks intervals during which no fluorescent spot was seen. Black and gray arrows designate episodes of forward and reverse unidirectional motions corresponding to the directions of sense and antisense transcription, respectively. Teal indicates intervals of RNAP^{549} random sliding. (**J**) Computing the position of retained RNAP^{549} on template DNA. Single-molecule emission records, as in Figures 1, 2, 3 and S1. The time

interval in the RNAP^{549} record indicated by the black arrow is fit to a single exponential decay model (solid blue curve, *Methods*). The dashed lines mark the time of termination t_T (red), the RNAP^{549} fluorescence intensity while bound to promoter I_P (green) and at the time of termination I_T (purple), and the mean background fluorescence when no RNAP^{549} spot is present I_{mn} (gray). Right, schematic of a model of RNAP^{549} position on the template DNA during constant-velocity elongation (*Methods*) accompanied by a depiction of template DNA indicating the approximate locations of the promoter (z_P) and terminator (z_T) regions. (**K**) Position of RNAP^{549} after termination calculated using the calibration depicted in (J) (*Methods*). Right, normalized histogram of RNAP^{549} position during the interval plotted at left.

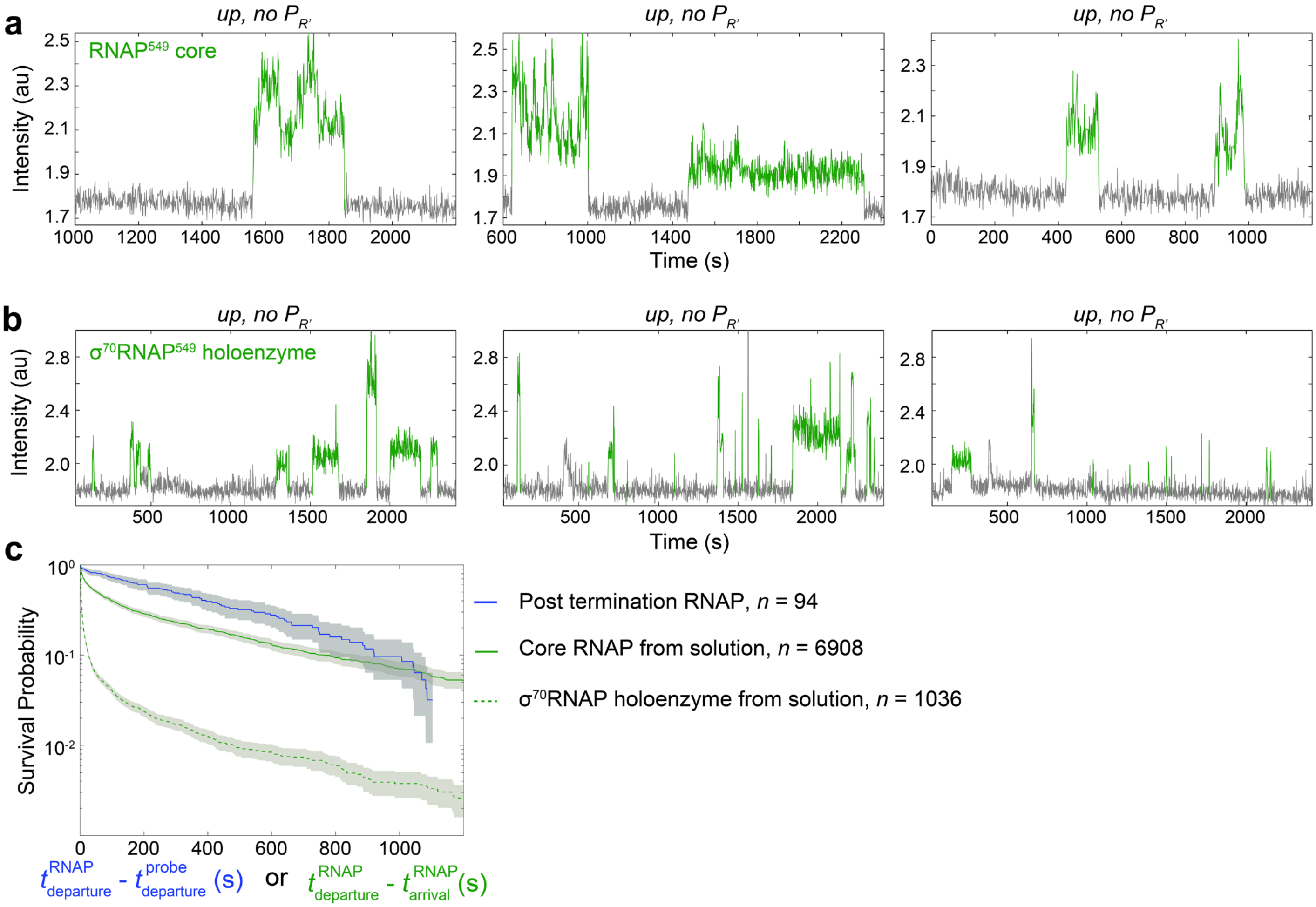

Supplementary Figure 3. Core RNAP or σ^{70}RNAP from solution binding to promoter-ablated template DNA molecules. (**A**) Example records illustrating core RNAP binding from solution and sliding on individual template DNA molecules that lack the $P_{R'}$ promoter sequence (*Methods*). Records are selected from an experiment in the absence of NTPs with 0.7 nM core RNAP^{549} introduced at time $t = 0$ showing RNAP^{549} emission colocalized with 3 different DNA spots. Gray color marks intervals during which no fluorescent spot was seen. (**B**) As in (A), but with 0.7 nM σ^{70}RNAP^{549} holoenzyme instead of core RNAP^{549}. (**C**) Survival curves of template DNA-bound RNAP^{549} (i.e., cumulative distributions of dwell times such as those shown as colored intervals in (A) and (B). Dwell times are taken from the experiments described in (A) (solid green), (B) (dashed green) and the blue subpopulation from Figure 1D, *top* (blue), which are the lifetimes of RNAP^{549} molecules retained on template DNA following termination. Shaded regions show the 90% confidence intervals of the curves determined by bootstrapping.

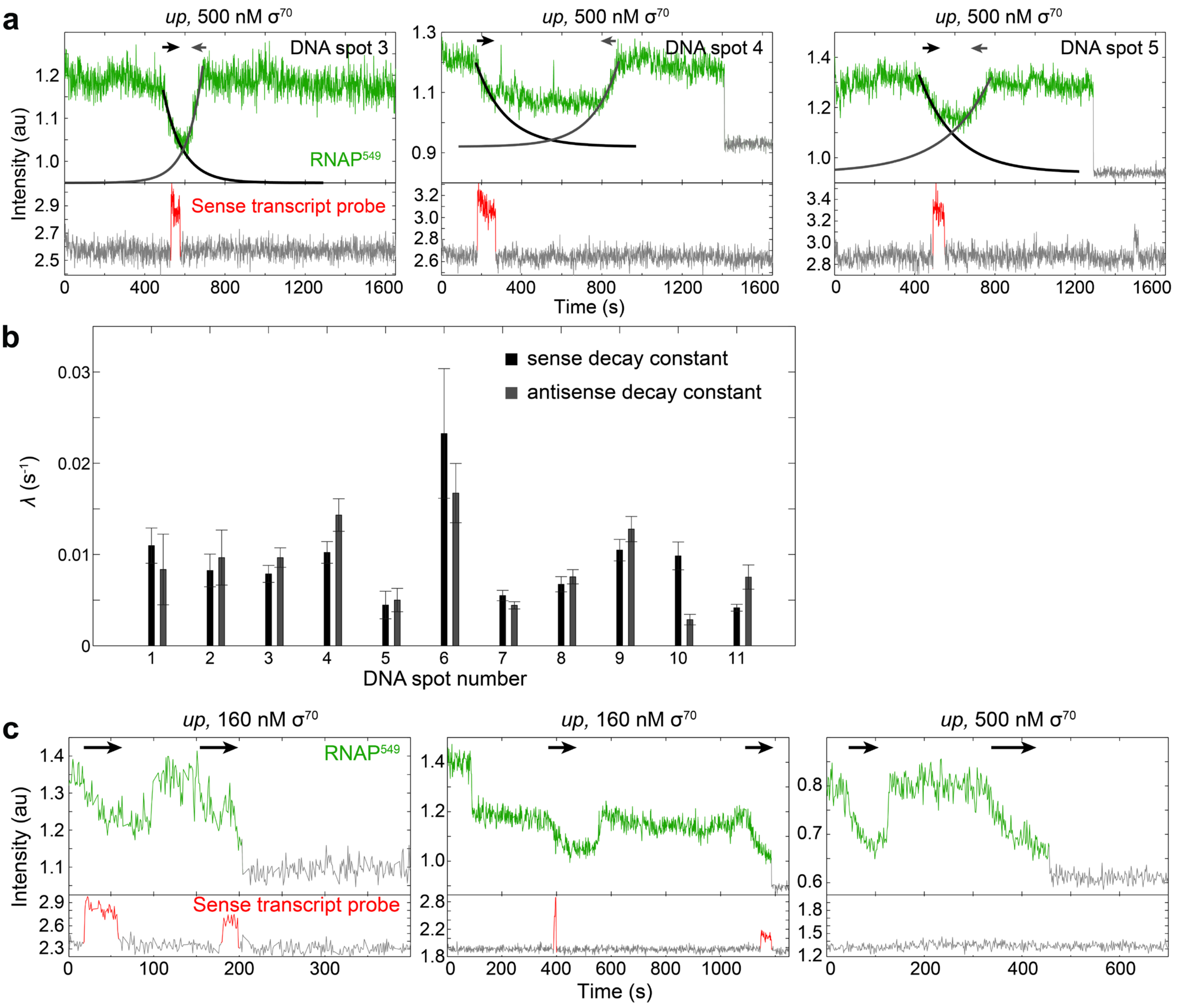

Supplementary Figure 4. Comparing the translocation speed of single RNAP^{549} molecules during sense and antisense transcription and example single-molecule fluorescence records indicative of multiple sense transcript initiations by the same RNAP molecule. (**A**) Single-molecule emission records, plotted as in Figure 3A, and fits to an exponential decay model (*Methods*) as in Supplementary Figure 2 for the sense (black) and antisense (gray) RNAP^{549} transcription signatures. (**B**) Comparison of exponential decay constants, λ (± s.e.m.) from fit curves like those depicted in (A) drawn from 11 randomly chosen fluorescence records that exhibit both sense (black) and antisense (gray) fluorescence signatures. (**C**) Plots show records selected from two different experiments illustrating the same molecular behavior as that in Figure 3E. Each plot shows RNAP^{549} and Cy5-transcript probe emission co-localized with 3 different DNA spots. Such traces are not typical, but rather show evidence that sense transcript re-initiation following RNAP^{549} sliding may infrequently take place. Occurrences like that of the example on the right, wherein apparent steady-state elongation is observed in the RNAP^{549} TFM signal without co-localized transcript probe signal are more frequent, comprising as much as 30% of observed RNAP^{549} TFM elongation signals; we interpret these as reflecting inefficient probe hybridization due to folding of the nascent transcript. Gray color marks intervals during which no fluorescent spot was detected.

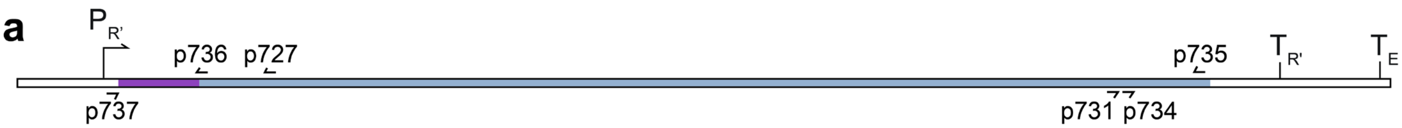

Primer		Sequence
p727	Sense cDNA primer	5'-CGGTTTCACCACGCAGGCGTT-3'
p731	Anti-sense cDNA primer	5'-GAAGACGAGATGTATCCGGGTGAA-3'
p734	Anti-sense upstream qPCR primer	5'-GGTATCGACATCTACAACCTGACCAA-3'
p735	Anti-sense downstream qPCR primer	5'-CGAAGTGACCAACTAGGCGGAAT-3'
p736	Sense upstream qPCR primer	5'-CAGAATGATGGTCGCAGGCAGTT-3'
p737	Sense downstream qPCR primer	5'-CGATGGGTTAATTCGCTCGGAT-3'

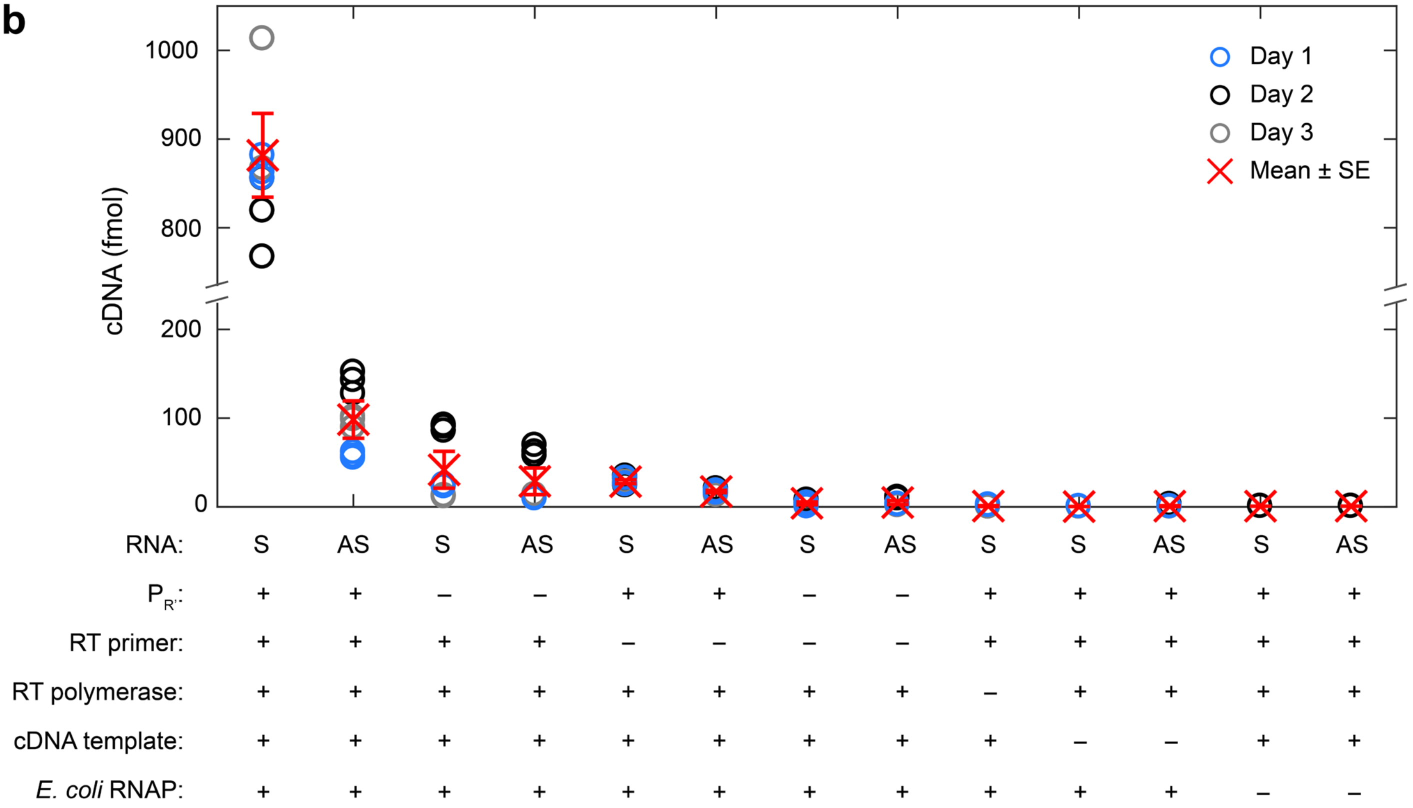

Supplementary Figure 5. Sense and antisense transcript production *in vitro* measured by RT-qPCR. (A) The transcription template and layout and sequences of the primers used for cDNA synthesis and qPCR. The template (same DNA sequence as that used in the single-molecule experiments) contains a wild type λ $P_{R'}$ promoter region (blue, bent arrow) followed by seven tandem repeats of a 21 bp cassette (maroon), most of the *E. coli rpoB* coding region (gray) and two consecutive intrinsic terminators, λ $T_{R'}$ and T7 T_E, respectively. **(B)** Full results of the RT-qPCR experiments shown in Figure 4, including control experiments that withheld a selected component of the RT-qPCR assay. Columns 1-4 correspond to the bars in Figure 4. Colored circles indicate triplicate qPCR measurements from each of three experimental replicates conducted on different days; × indicates mean ± SE (n = 3). Samples designated minus $P_{R'}$ used a version of the template in which promoter sequences were mutated (see *Methods*).

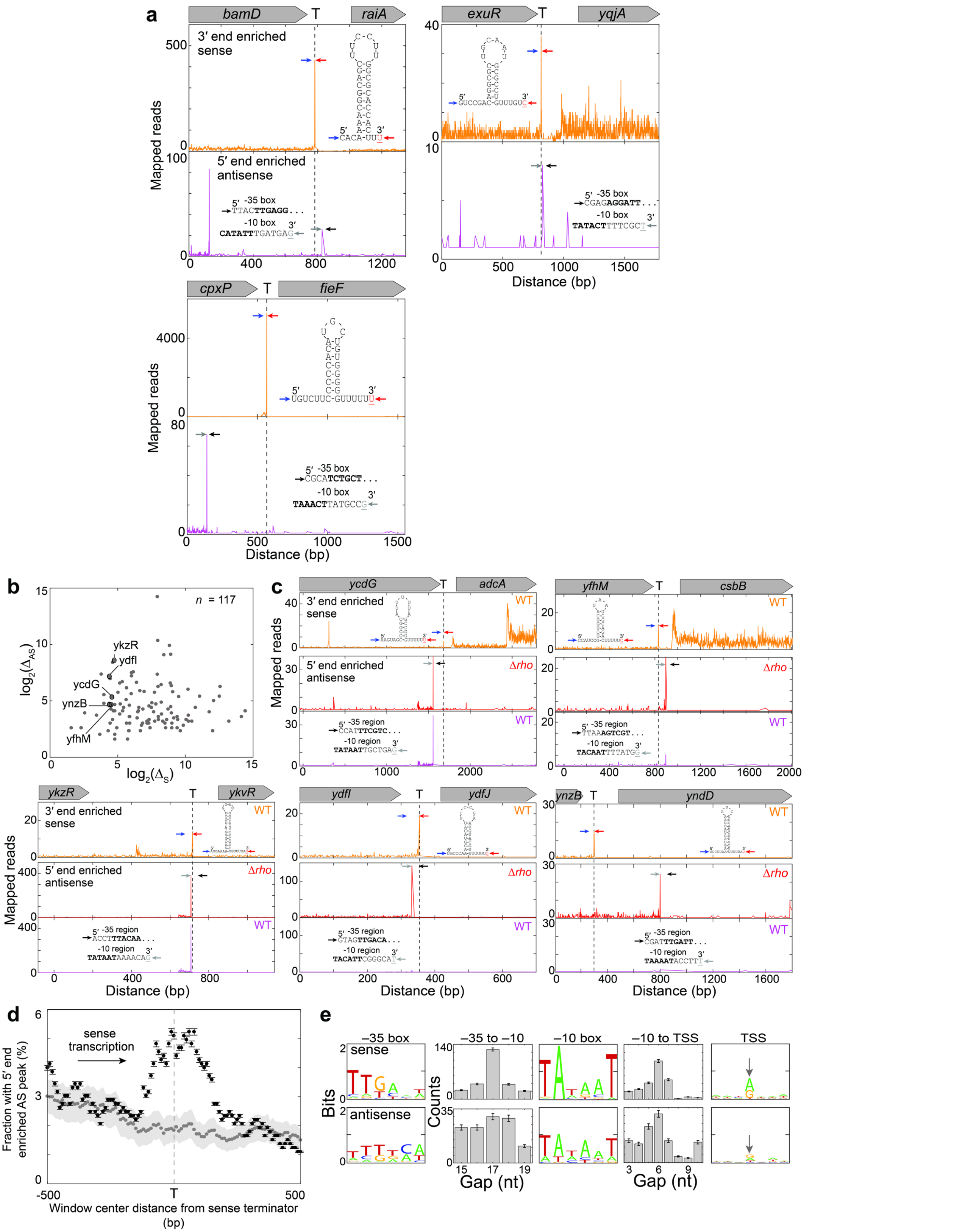

Supplementary Figure 6. Evidence in *E. coli* and *B. subtilis* Rend-seq data for secondary initiation of antisense transcripts near positions of sense terminators. (**A**) Data from three example *E. coli* terminators

chosen from Figure 5B and plotted as in Figure 5C. The 3′ end of each sense RNA exhibits the step-loop structure and U-rich tract characteristic of an intrinsic (i.e., Rho-independent) terminator. (**B**) Rend-seq peak heights from a Δ*rho* *B. subtilis* strain, computed and plotted as in Figure 5B, for 117 of 726 terminators between genes transcribed in the same direction (see *Methods*) that show a substantial Δ_{AS} peak within 500 nt of the terminator Δ_{S} peak. The mutant strain is used because the Δ*rho* mutation in *B. subtilis* increases the steady-state levels of antisense transcripts, making them easier to detect and measure[4–6]. (**C**) Data from five example terminators chosen from (B) and plotted as in (A). Antisense data from both wild-type (WT) and Δ*rho* strains are shown; the WT data set was normalized to have the same total reads as the Δ*rho* data set. (**D**) Antisense (AS) initiation peak frequency correlates with positions of sense terminators in the *B. subtilis* genome. Pooled data (Δ*rho* strain) from 726 terminators between genes transcribed in the same direction (see *Methods*). Data are analyzed and plotted as in Figure 5D. Plot shows the fraction (± s.e.m.) of 200 nt-wide windows centered at the indicated distance upstream or downstream from the terminators that exhibit a peak of antisense initiation (black). Also shown is the mean ± SD of negative controls (gray) in which the same analysis was repeated 100 times using 726 randomly selected locations in the *B. subtilis* genome that lack an apparent terminator. In 100% of these 100 control replicates, the fraction at the terminator location with a 5′ end AS peak was < 2.8%, indicating that the difference between experimental data and controls was significant ($p < 10^{-2}$). (**E**) Sequence characteristics (illustrated as in Figure 5E) for n = 250 strong sense *B. subtilis* promoters (*top*) and for the n = 117 terminator-proximal antisense initiation sites shown in (B) (*bottom*).

SUPPLEMENTARY TABLE

Supplementary Table 1. Oligonucleotide sequences.

Oligonucleotide Name	Sequence (5´ to 3´)
p397	biotin-CCTATAAAAATAGGCGTATCACGAG
p447	AlexaFluor 488-AGATATCGCAGAAAGGCCCACCCGAAGGTG AGCCAGTGTGATTACCAGGGTTTTCCCAGTCACGACCTT
p727	CGGTTTCACCACGCAGGCGTT
p731	GAAGACGAGATGTATCCGGGTGAA
p734	GGTATCGACATCTACAACCTGACCAA
p735	CGAAGTGACCAACTAGGCGGAAT
p736	CAGAATGATGGTCGCAGGCAGTT
p737	CGATGGGTTAATTCGCTCGGAT
Sense probe	GTGTGTGGTCTGTGGTGTCT-Cy5
Antisense probe	AGACACCACAGACCACACAC-Cy5

SUPPLEMENTARY REFERENCES

1. Harden, T. T. *et al.* Bacterial RNA polymerase can retain σ^{70} throughout transcription. *Proceedings of the National Academy of Sciences of the United States of America* **113**, 602–607 (2016).
2. May, P. F. J. *et al.* Tethered Fluorophore Motion: Studying Large DNA Conformational Changes by Single-fluorophore Imaging. *Biophysical Journal* **107**, 1205–1216 (2014).
3. Friedman, L. J. & Gelles, J. Multi-wavelength single-molecule fluorescence analysis of transcription mechanisms. *Methods* **86**, 27–36 (2015).
4. Bidnenko, V. *et al.* Termination factor Rho: From the control of pervasive transcription to cell fate determination in *Bacillus subtilis*. *PLoS Genetics* **13**, (2017).
5. Lalanne, J.-B. *et al.* Evolutionary Convergence of Pathway-Specific Enzyme Expression Stoichiometry. *Cell* **173**, 749–761 (2018).
6. Nicolas, P. *et al.* Condition-Dependent Transcriptome Architecture in *Bacillus subtilis*. *Science* **335**, 1103–1106 (2012).
