# Supplementary material for: Alternative transcription cycle for bacterial RNA polymerase
Source: Nat Commun. 2020 Jan 23;11:448. doi: 10.1038/s41467-019-14208-9 (PMC6978322; doi:10.1038/s41467-019-14208-9)
Supplement: Supplementary file 2 — Peer Review File [file 41467_2019_14208_MOESM2_ESM.pdf]

Reviewers' comments:

Reviewer #1 (Remarks to the Author):

The authors present the results of single molecule fluorescence and transcript sequencing to make the case that most of the time, RNAP does not dissociate after termination and dissociation of transcript. Instead RNAP enters a 1D diffusional state where it scans the double-stranded DNA for promoter-like motifs and is capable of re-initiation events on both template and non-template strands. This observation is in contrast to dogma and is quite interesting and potentially significant and, as such, appropriate for publication in Nat Comm. However, I have a few questions that should be addressed prior to publication:

(1) What is the salt-concentration dependence of this behavior? Current experiments are carried out at 100mM KAc. Do higher salt concentrations lead preferentially to RNAP dissociation.

(2) In Figure 1C RNAP appears bound indefinitely. Is what percentage of the traces does this occur? Does it eventually dissociate or is it STUCK somehow?

(3) Are AS initiation events observed in the absence of Pr? The authors show by qPCR that the amount of AS is linked to the presence of the sense promoter, but is there something that prevents the initiation of the antisense transcription de novo? Seems like the model predicts that in the absence of Pr, rare examples of AS transcription should be seen in the single-molecule assay, perhaps with higher polymerase concentration?

(4) What about on supercoiled templates in vitro? Is the secondary transcript phenomenon still observed? Also, if RNAP concentration is increased, does the secondary transcript lose its dependence on the primary promoter suggesting the ability of RNAP to interact weakly de novo with these sequences?

(5) Sequence results suggest that there is a slight enrichment of promoter-like sequences within a short distance of terminators, but this does not say anything directly regarding re-initiation as caveated by the authors themselves. This however could be tested in vivo as was done in vitro by mutating the “primary promoter” and measuring the “secondary promoter” in vivo (a suggestion; not necessarily a requirement for publication)

(6) In the discussion, the proposed mechanism of antisense via the same polymerase seems specious. After production of a transcript (i.e. gene is on), the subsequent reverse sense transcript by the same enzyme would likely create head-on collisions between polymerases. Would these result in stalled complexes, or can two polymerases pass one another if they are using different strands? I believe this same mechanism is actually invoked by the authors to explain the different ratios of sense/antisense in the RT-PCR experiment.

(7) In Fig3A, up, no sig70, why does antisense transcription occur? Is the model that this is due a retained sigma factor?

(8) Why are some signal changes on the order of 2 a.u. and others 0.2 a.u? Fig1C, Fig2A, FigS1D, FigS1F, FigS2A-I? Does this have to do with the location of the spot in the field of view?

(9) In the description of the assay, they say that "RNAP is incubated with the surface". I suggest this be changed to "incubated the flow cell" as it otherwise sounds like the RNAP should be interacting with the surface directly.

(10) The data presented indicate that a de novo bound polymerase (core or holo) has different properties compared to one that has entered the diffusive state after termination of a primary

transcript. In particular, it interact with dsDNA with a much longer lifetime and exhibits 1D diffusion. I would ask the authors to speculate on the potential structural differences between these kinds of polymerases that may lead to these differences.

Reviewer #2 (Remarks to the Author):

In this work, Harden et al. used single-molecule fluorescence imaging to study the bacterial transcription cycles. They made the surprising finding that RNAP remains bound to DNA after termination and RNA release, and that the same RNAP is able to re-initiate a second round of transcription, often producing an antisense transcript. They also provided compelling in vivo evidence that these alternative pathways are indeed functional inside the cell. This is an excellent study that builds upon the same group's long-standing expertise in using single-molecule fluorescence to investigate bacterial transcription. The power of multi-color CosMos was utilized to its full potential. Many well-designed controls make the single-molecule results almost impeccable (with a few exceptions as detailed below). This work provides a potentially paradigm-shifting contribution to the understanding of transcription mechanisms. As such, I believe it will be well-received by the broad readership of Nature Communications. I recommend this manuscript for publication after a few minor points are addressed.

(1) Transcription termination is scored upon the loss of Cy5 signal. The authors justify this interpretation by stating that under the experimental conditions, photobleaching is not significant and therefore loss of signal most likely corresponds to termination. To support this claim, the reader is directed to Fig. S1A which demonstrates that in a previous study from the same group, Cy3-transcript probe lifetimes were unaffected by different fractions of laser exposure. However, the authors only present a single Cy5-transcript probe lifetime histogram in this study. Given the similar experimental setups between studies, I do believe that Cy5-transcript loss is reporting on termination. However, comparison of photobleaching rates of Cy3 vs Cy5 is not an ideal method to differentiate photobleaching from biological activity. The authors should either 1) perform an additional Cy5-lifetime control under a different fractional exposure or 2) clarify that the lifetime of the process is known from the 2016 paper, and therefore the similar Cy5-probe result in this study reports on termination rather than photobleaching.

(2) While I agree with the authors' interpretation and analysis of RNAP sliding dynamics, the single-molecule method used in this work does not unambiguously distinguish sliding from other interpretations for the observed intensity fluctuations, such as protein induced fluorescence enhancement (PIFE). The authors should comment on these alternative explanations, and why they are disfavored. While not required for publication, a shorter DNA template could be used to rule out post-termination conformational dynamics (PIFE) vs. sliding.

(3) The authors compare the behavior of post-termination RNAP in the presence of free sigma70 to that in its absence, and conclude that the sliding RNAP is the core complex rather than the holoenzyme. The group does have the capability to monitor the co-localization of RNAP and sigma70 as demonstrated in prior publications. This experiment should be performed in order to determine the identities of the sliding complex and the unidirectional complex. If it cannot be done for technical reasons, those difficulties should be explained.

(4) The probes to detect antisense transcription in the single-molecule assay and in the RT-qPCR assay are targeting different regions of the template, making their comparison (31% vs. 11%) less direct. Can the probe in one of these assays be changed to match the other? Regarding the the single-molecule assay (Figure 3C), the antisense RNA seems to be released in all example traces. But there is no intrinsic terminator in the reverse direction? Please clarify.

(5) The authors did not explain why they chose the Rho-deletion strain of B. subtilis to analyze the correlation between sense 3’ ends and antisense 5’ ends (Figure S6B). Is it because the correlation is weaker in the wildtype strain? If so, does it imply that the RNAP recycling mechanism plays a lesser role in B. subtilis than in E. coli? How does this relate to the differential essentiality of Rho in these bacteria? The 3’ end sense data for the delta-Rho strain should be provided in Figure S6C for direct comparison. While the genomic analysis was mainly used as supporting evidence in this paper, more explanation and discussion in this part would help the readers to appropriately evaluate the potential importance of RNAP recycling in vivo.

(6) In the Discussion, the authors outlined the precedence for enzyme flipping. Given that secondary antisense initiation was found to occur more frequently than secondary sense initiation in the single-molecule data of this work, RNAP flipping seems to be just as prevalent as if not more than long-distance sliding. Does flipping happen stochastically at all locations, or does it prefer certain sites (e.g., upstream or downstream of a terminator)? The authors may want to speculate on these points and suggest future experiments to directly probe the flipping dynamics.

Verbatim reviewers' comments (in black) and author responses (in blue).

Reviewer #1 (Remarks to the Author):

The authors present the results of single molecule fluorescence and transcript sequencing to make the case that most of the time, RNAP does not dissociate after termination and dissociation of transcript. Instead RNAP enters a 1D diffusional state where it scans the double-stranded DNA for promoter-like motifs and is capable of re-initiation events on both template and non-template strands. This observation is in contrast to dogma and is quite interesting and potentially significant and, as such, appropriate for publication in Nat Comm. However, I have a few questions that should be addressed prior to publication:

(1) What is the salt-concentration dependence of this behavior? Current experiments are carried out at 100mM KAc. Do higher salt concentrations lead preferentially to RNAP dissociation.

As stated in the manuscript, the ionic conditions we used are designed to mimic physiological conditions. While the effect of higher salt might be interesting to explore in future work, we feel that such investigation is beyond the scope of the present manuscript and is not necessary to support the conclusions we reach.

(2) In Figure 1C RNAP appears bound indefinitely. Is what percentage of the traces does this occur? Does it eventually dissociate or is it STUCK somehow?

The fraction of molecules that never display the freely sliding behavior after termination is one minus the sliding fraction shown as blue bars in Fig. 3B. For molecules that appeared immobile until the fluorescent spot disappeared, we cannot tell whether disappearance is because the RNAP dissociated from DNA or because the dye attached to the RNAP photobleached and the RNAP remained stuck. Heparin is a polymer that competes with DNA for binding to RNAP. The absence of such post-termination "stuck" molecules when heparin is added (Fig. 1D) is consistent with the idea that such molecules are stuck to DNA, not to the slide surface.

(3) Are AS initiation events observed in the absence of Pr?

The qPCR experiment (Fig. 4) shows clearly that a small amount of AS transcript is produced on DNA with an ablated sense promoter. The analogous single-molecule experiment is not feasible because the single molecule protocol requires pre-forming an open complex and washing out free RNAP from the chamber. This cannot be done without the sense promoter since most holoenzyme molecules have only a very short lifetime (~10 s) on DNA with an ablated sense promoter (Fig. S3C).

The authors show by qPCR that the amount of AS is linked to the presence of the sense promoter, but is there something that prevents the initiation of the antisense transcription de novo? Seems like the model predicts that in the absence of Pr, rare examples of AS transcription should be seen in the single-molecule assay, perhaps with higher polymerase concentration?

We specifically avoid calling AS initiation site a promoter (we call it “promoter-like”) because it may not function by the normal mechanism (recruiting RNAP from solution). Instead, it may only work well in cases where RNAP already bound to DNA. We explicitly discuss this possibility in the manuscript (see last paragraph of Results section). Since we are cognizant of this possibility, we do *not* predict recruitment of RNAP from solution to the AS promoter in the model as proposed (Fig. 6). The single-molecule experiment is not technically feasible at significantly higher concentrations of σ^{70}RNAP.

(4) What about on supercoiled templates in vitro? Is the secondary transcript phenomenon still observed?

The single-molecule experiments require linear DNA. We do not have the technical capability to follow the motion of RNAP on circular DNA that can be supercoiled. We have tried the RT-qPCR experiments on plasmids containing the transcription template sequences. These experiments did not produce interpretable results, we think because of sequestration of RNAP by the antibiotic resistance gene transcription unit.

Also, if RNAP concentration is increased, does the secondary transcript lose its dependence on the primary promoter suggesting the ability of RNAP to interact weakly de novo with these sequences?

We are assuming this comment refers to the RT-qPCR experiment and that the “sequences” mentioned refer to the AS initiation sites. RT-qPCR was already done with RNAP at 1:1 molar stoichiometry with DNA. It is not feasible to substantially increase the concentrations; in our hands working at higher RNAP concentrations causes DNA condensation which inhibits transcription.

(5) Sequence results suggest that there is a slight enrichment of promoter-like sequences within a short distance of terminators, but this does not say anything directly regarding re-initiation as caveated by the authors themselves. This however could be tested in vivo as was done in vitro by mutating the “primary promoter” and measuring the “secondary promoter” in vivo (a suggestion; not necessarily a requirement for publication)

We appreciate the suggestion and may conduct this experiment in future work. Non-coding transcripts in bacteria are prematurely terminated and degraded in processes that are very sensitive to small perturbations of RNA structure. Thus, while experiments of this type are feasible, they are non-trivial to interpret without extensive controls. Such work is beyond the scope of the present paper.

(6) In the discussion, the proposed mechanism of antisense via the same polymerase seems specious. After production of a transcript (i.e. gene is on), the subsequent reverse sense transcript by the same enzyme would likely create head-on collisions between polymerases. Would these result in stalled complexes, or can two polymerases pass one another if they are using different

strands? I believe this same mechanism is actually invoked by the authors to explain the different ratios of sense/antisense in the RT-PCR experiment.

There are numerous, well established examples by which antisense transcription regulates bacterial gene expression (reviewed in refs. 10 and 17; these are cited in this context in the last paragraph of the Discussion section). Some known examples of antisense regulation work by hybridization of S and AS RNAs, while others (termed “transcriptional interference”) are mediated by polymerase collisions. Since such collisions are *already established as a regulatory mechanism*, we do not regard the possibility of collisions as a weakness of our hypothesized mechanism. Collisions have been shown to have multiple different outcomes as discussed in the cited references.

(7) In Fig3A, up, no sig70, why does antisense transcription occur? Is the model that this is due a retained sigma factor?

Yes. In the revised manuscript we say this explicitly in the Discussion section. This phenomenon is reproducible but rare (<5%; see leftmost gray bar in Fig. 3B).

(8) Why are some signal changes on the order of 2 a.u. and others 0.2 a.u? Fig1C, Fig2A, FigS1D, FigS1F, FigS2A-I? Does this have to do with the location of the spot in the field of view?

In the manuscript fluorescence intensities are expressed in arbitrary units. Reported intensity values between different experimental conditions in some cases differed by an arbitrary normalization factor; that accounted for the ~10-fold difference between records in different figures noted by the reviewer. To avoid confusion, we have modified the figures so that the same, constant scaling factor is used for all conditions and across all figures. The reviewer is correct in that there is some variation in single-dye intensity between different locations in the microscope field of view, and this likely accounts for any remaining record-by-record differences in signal amplitude.

(9) In the description of the assay, they say that "RNAP is incubated with the surface". I suggest this be changed to "incubated the flow cell" as it otherwise sounds like the RNAP should be interacting with the surface directly.

We made this change. Thank you for the suggestion.

(10) The data presented indicate that a de novo bound polymerase (core or holo) has different properties compared to one that has entered the diffusive state after termination of a primary transcript. In particular, it interact with dsDNA with a much longer lifetime and exhibits 1D diffusion. I would ask the authors to speculate on the potential structural differences between these kinds of polymerases that may lead to these differences.

In our view, the data show that de novo bound core RNAP behaves *almost identically* to the post-termination RNAP. In both cases, the majority population of molecules makes stable

sliding interactions with DNA (Fig. S3A and S3C). De novo bound core contains an additional subpopulation (~45%) that displays a characteristic shorter lifetime; this subpopulation is not present in the post-termination RNAP (Fig. S3C). These short lifetime complexes undoubtedly have a different structure than do the long-lived ones, but we know of no basis for speculation as to the details of the structural difference.

In contrast, >90% of de novo holoenzyme binding events show much shorter lifetimes (Fig. S3C) and little or no detectable sliding (Fig. S3B). The small (<10%) component of the lifetime distribution with a longer characteristic lifetime might be caused by residual core RNAP present in the reconstituted σ^{70}RNAP preparation.

We have clarified discussion of the above points in the manuscript.

Reviewer #2 (Remarks to the Author):

In this work, Harden et al. used single-molecule fluorescence imaging to study the bacterial transcription cycles. They made the surprising finding that RNAP remains bound to DNA after termination and RNA release, and that the same RNAP is able to re-initiate a second round of transcription, often producing an antisense transcript. They also provided compelling in vivo evidence that these alternative pathways are indeed functional inside the cell. This is an excellent study that builds upon the same group's long-standing expertise in using single-molecule fluorescence to investigate bacterial transcription. The power of multi-color CosMos was utilized to its full potential. Many well-designed controls make the single-molecule results almost impeccable (with a few exceptions as detailed below). This work provides a potentially paradigm-shifting contribution to the understanding of transcription mechanisms. As such, I believe it will be well-received by the broad readership of Nature Communications. I recommend this manuscript for publication after a few minor points are addressed.

We thank the reviewer for this favorable evaluation of the work and we address below the minor points raised.

(1) Transcription termination is scored upon the loss of Cy5 signal. The authors justify this interpretation by stating that under the experimental conditions, photobleaching is not significant and therefore loss of signal most likely corresponds to termination. To support this claim, the reader is directed to Fig. S1A which demonstrates that in a previous study from the same group, Cy3-transcript probe lifetimes were unaffected by different fractions of laser exposure. However, the authors only present a single Cy5-transcript probe lifetime histogram in this study. Given the similar experimental setups between studies, I do believe that Cy5-transcript loss is reporting on termination. However, comparison of photobleaching rates of Cy3 vs Cy5 is not an ideal method to differentiate photobleaching from biological activity. The authors should either 1) perform an additional Cy5-lifetime control under a different fractional exposure or 2) clarify that the lifetime of the process is known from the 2016 paper, and therefore the similar Cy5-probe result in this study reports on termination rather than photobleaching.

Yes, our intended explanation was #2. We have revised the Fig. S1 caption to make this clear.

(2) While I agree with the authors' interpretation and analysis of RNAP sliding dynamics, the single-molecule method used in this work does not unambiguously distinguish sliding from other interpretations for the observed intensity fluctuations, such as protein induced fluorescence enhancement (PIFE). The authors should comment on these alternative explanations, and why they are disfavored. While not required for publication, a shorter DNA template could be used to rule out post-termination conformational dynamics (PIFE) vs. sliding.

The reviewer agrees with our interpretation [intensity fluctuations reflect differences in tethered fluorophore motion (TFM), which report sliding] but asks about the possibility of alternative interpretations. Only a single alternative, PIFE, is mentioned. PIFE is fluorescence enhancement of a dye attached to DNA (or RNA) by an unlabeled protein, and PIFE depends on close (sub-nanometer) approach of the protein to the dye that affects dye conformation via direct protein-dye contact [Hwang, H. & Myong, S. Chem. Soc. Rev. 43, 1221–1229 (2014)]. In contrast, we here are observing a dye attached to RNAP protein (unlike reported PIFE), and it is attached at a location unlikely to contact DNA. We cannot unambiguously exclude the involvement of some other previously unreported fluorescence enhancement/quenching phenomenon that has unspecified characteristics, but such an explanation appears unlikely. The magnitude of the fluorescence intensity fluctuations corresponds to that seen for movement over the micrometer-scale length of DNA during transcription; those changes during transcription are caused by TFM as confirmed by changes in fluorescence spot width (Fig. S1e). (Enhancement/quenching alone would not change spot width.) The intensity changes seen during sliding are graded, not all-or-nothing (e.g., Fig. S2j, green) and, when interpreted as TFM, have dynamics consistent with bounded diffusion over approximately the full length of the DNA (Fig. 2b, blue). Thus, the most straightforward explanation is that the intensity fluctuations in question are caused by the same TFM phenomena that we have validated as occurring during transcript elongation.

(3) The authors compare the behavior of post-termination RNAP in the presence of free sigma70 to that in its absence, and conclude that the sliding RNAP is the core complex rather than the holoenzyme. The group does have the capability to monitor the co-localization of RNAP and sigma70 as demonstrated in prior publications. This experiment should be performed in order to determine the identities of the sliding complex and the unidirectional complex. If it cannot be done for technical reasons, those difficulties should be explained.

We appreciate the reviewer's confidence in our technical capabilities, but in fact we have no prior publications in which we monitored co-localization of σ^{70} with RNAP. Such experiments are technically challenging for a variety of reasons. These reasons include that not every molecule of either protein is labeled so that deriving conclusions is inherently statistical and thus requires large numbers of observations.

We have attempted the experiment proposed by the reviewer twice, trying two different pairs of dye labels on σ^{70} and RNAP. In neither case did we obtain a sufficient number of triply-labeled (σ^{70} / RNAP / nascent RNA) elongation complexes to allow for clear conclusions about the presence or absence of σ^{70} on sliding RNAPs or on the tiny fraction (~ 5%; see Fig. 3B) of

post-termination complexes, or about the presence or absence of σ^{70} on complexes that displayed reverse motion (i.e., secondary initiation and AS transcription) in the absence of added σ^{70} in solution. (The experiment is not feasible with labelled σ^{70} in solution because the high concentrations required would preclude single-molecule detection of the protein in the transcription complexes.)

Despite the above, we emphasize that the manuscript and our previous work already contains information on the capabilities of RNAP vs. σ^{70}RNAP in producing sliding and unidirectional motion. This information includes:

- Only ~25% of complexes retain σ^{70} at the terminator whereas ~45% slide in the absence of added σ^{70} (Fig. 1B). Thus, complexes without σ^{70} are capable of sliding.
- Binding of σ^{70}RNAP is mostly too transient (Fig. S3) to determine using our methods whether or not it slides (we have added text to the manuscript to say this explicitly).
- Our previous work [Harden, T. T. et al. PNAS 113, 602–7 (2016)] shows that both RNAP and σ^{70}RNAP can form active elongation complexes and are therefore capable of unidirectional motion.

(4) The probes to detect antisense transcription in the single-molecule assay and in the RT-qPCR assay are targeting different regions of the template, making their comparison (31% vs. 11%) less direct. Can the probe in one of these assays be changed to match the other?

The single-molecule experiment uses a probe that targets multiple direct repeats of a target sequence in the RNA. It is not technically feasible to do the single-molecule experiment using the RT probes, which have only a single target sequence and are not optimized for fast binding kinetics, because these differences would likely make nascent transcript detection too slow/inefficient to be usable. Conversely, using the single-molecule probes for RT-qPCR would considerably complicate interpretation of that experiment because they would allow initiation of multiple closely spaced reverse transcriptase complexes on the same RNA.

Regarding the the single-molecule assay (Figure 3C), the antisense RNA seems to be released in all example traces. But there is no intrinsic terminator in the reverse direction? Please clarify.

We added to the manuscript the following comment on this point: “Consistent with the absence of an antisense terminator sequence in the template, the antisense probe fluorescence was often retained for hundreds of seconds after the reverse motion completed (e.g., Figure 3C). Eventual disappearance of antisense probe fluorescence could be explained by dissociation of the run-off transcription complex, probe dissociation from transcript, and/or photobleaching.”

(5) The authors did not explain why they chose the Rho-deletion strain of B. subtilis to analyze the correlation between sense 3’ ends and antisense 5’ ends (Figure S6B). Is it because the correlation is weaker in the wildtype strain? If so, does it imply that the RNAP recycling mechanism plays a lesser role in B. subtilis than in E. coli?

We explained in the manuscript that in vivo "…steady-state levels of antisense transcripts are greatly increased by Rho mutation or inhibition[39–42]." This effect is thought (see cited refs.) to arise because Rho activity preferentially targets AS transcripts and other products of pervasive transcription, causing those RNAs to be shorter and more susceptible to exonucleolytic degradation. Enhanced degradation and earlier termination reduce detectability of these RNAs by sequencing methods. We have now added text to the Fig. S6B caption that explicitly says the increased detectability of AS transcripts is why the Rho-deletion strain was used for the data in that panel. Note that the same analysis was done with similar results for *wild-type* E. coli in Fig. 5B, so the results are clearly not dependent on the absence of Rho activity, at least not in that species. We see no basis in any of our data for conclusions about the relative importance of the proposed mechanism in the two species.

How does this relate to the differential essentiality of Rho in these bacteria?

We are not aware of any such relationship. As indicated above, we see evidence for recycling whether or not Rho is present. We simply do not think that our data speaks to the question of why Rho is essential in E. coli and not in B. subtilis, which is in any case beyond the scope of the manuscript.

The 3' end sense data for the delta-Rho strain should be provided in Figure S6C for direct comparison.

The intent of including the 3' end sense data in Fig. S6C is to show examples of the termination data from the *wild-type* strain that were used to identify the population of 1,486 B. subtilis terminators (see Methods and ref. 39) that was used as the starting point for analysis of *both* wild type and Δ*rho* B. subtilis strains. As mentioned above, analysis of the Δ*rho* strain is included in the manuscript not because we are trying to determine the effects of Rho, but only because AS RNA initiation events (e.g., those seen in "5'-end enriched antisense" data in Fig. S6C) are more uniformly detectable in this genetic background. Thus, while we are providing the requested data for the reviewer's perusal (see figures at the end of this document), we are reluctant to include these additional panels in Fig. S6 for two reasons: 1) we do not want readers to be distracted by comparing the results of the 3'-end enriched data; there are many reasons why these data may exhibit minor differences between the two strains but these differences are irrelevant to the conclusions we derive from the figure (in S6D and S6E); and 2) we want to avoid adding complexity to what is already a large and complicated figure. We have added text to the Fig. S6 caption noting that the **sense termination sites analyzed are at intrinsic (i.e., Rho-independent) terminator sequences**.

While the genomic analysis was mainly used as supporting evidence in this paper, more explanation and discussion in this part would help the readers to appropriately evaluate the potential importance of RNAP recycling in vivo.

We share the reviewer's interest in the analysis of the Rend-seq data. The manuscript already includes explanation and discussion of this data in the last subsection of the Results section and

in the last paragraph of the Discussion section. In the revised manuscript we have edited this text to clarify. We have no additional points of explanation/discussion that we feel justified in adding, but we would be happy to consider any specific suggestions for additions.

(6) In the Discussion, the authors outlined the precedence for enzyme flipping. Given that secondary antisense initiation was found to occur more frequently than secondary sense initiation in the single-molecule data of this work, RNAP flipping seems to be just as prevalent as if not more than long-distance sliding. Does flipping happen stochastically at all locations, or does it prefer certain sites (e.g., upstream or downstream of a terminator)? The authors may want to speculate on these points and suggest future experiments to directly probe the flipping dynamics.

We do not have any evidence that the likelihood of flipping varies across a given DNA sequence. The relative prevalence of S vs. AS initiation in our experiments, while dependent on the kinetics of sliding and flipping (as the reviewer suggests) is also expected to depend strongly on the locations and strengths of S and AS promoter(-like) sequences on the DNA. The data in our paper do not permit evaluation of the relative contributions of these different factors. We would prefer not to speculate on this point until relevant evidence is available.

Data for Reviewer 2 comment 5: The five pairs of graphs shown on the following pages present the requested data. For the five example terminators shown in Figure S6C, we here show (in **blue**) the Rend-seq **3´ end-enriched sense** data from the Δ*rho* strain. For the reviewer's convenience, we also show (in **red**) the **5' end-enriched antisense** data from the Δ*rho* strain – this is the same dataset shown in the red plots in Fig. S6C. Orientation of genome segments is identical to those shown in Figure S6C (i.e., sense RNA is transcribed left-to-right in all cases) Black "+" shows the positions of the intrinsic terminator sequence (the same position that is marked "T" in Fig. S6C). Green "+" shows the inferred position of the antisense transcription start site. For all graphs the axes are the same as in Fig. S6C: horizontal – Distance (bp); vertical – Mapped Reads.

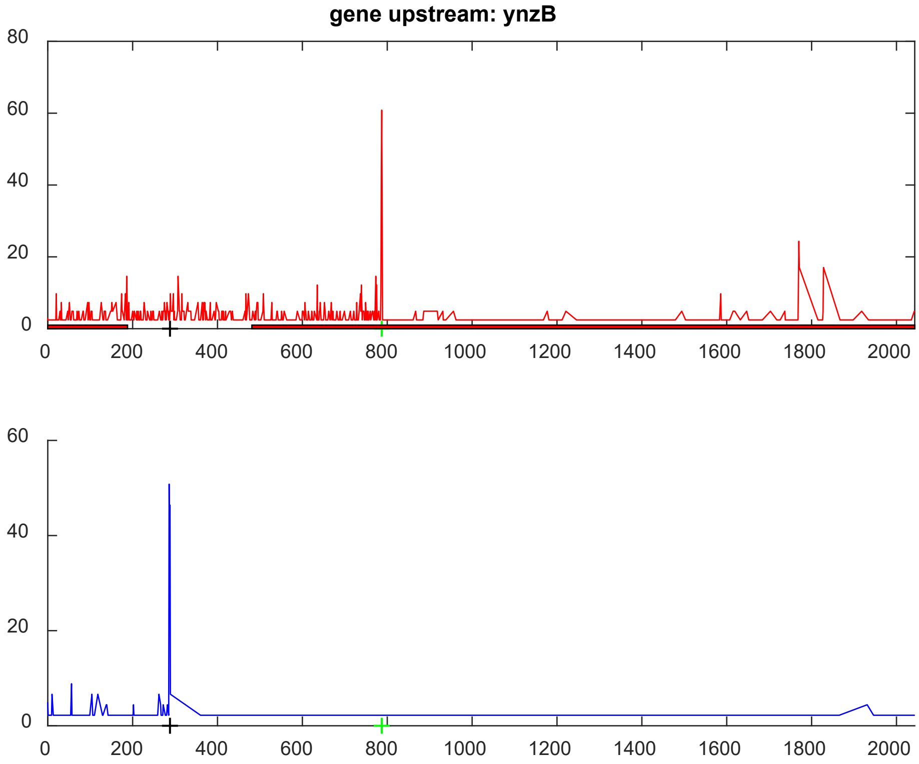
gene upstream: ynzB
80
60
40
20
0
0
200
400
600
800
1000
1200
1400
1600
1800
2000
60
40
20
0
0
200
400
600
800
1000
1200
1400
1600
1800
2000

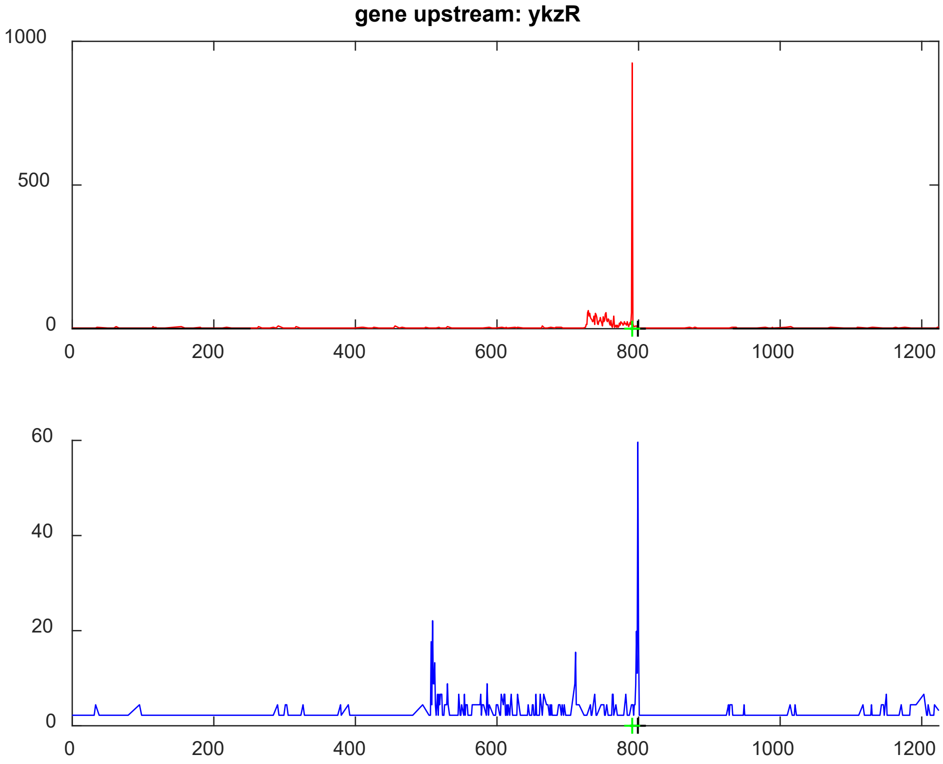
gene upstream: ykzR
1000
500
0
0
200
400
600
800
1000
1200
60
40
20
0
0
200
400
600
800
1000
1200

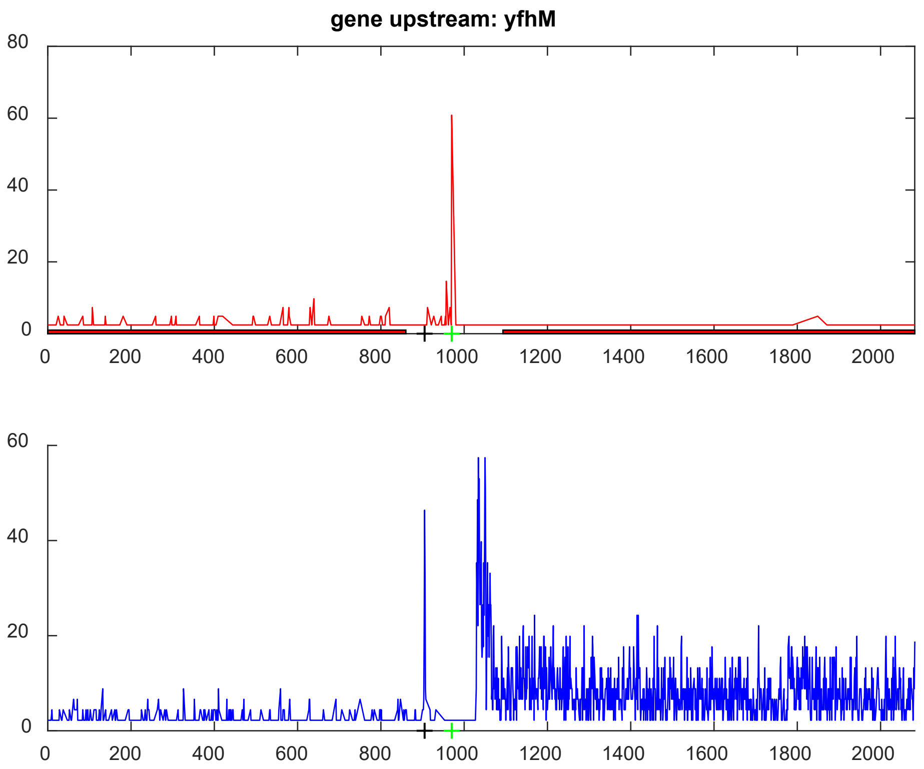
gene upstream: yfhM
80
60
40
20
0
0
200
400
600
800
1000
1200
1400
1600
1800
2000
60
40
20
0
0
200
400
600
800
1000
1200
1400
1600
1800
2000

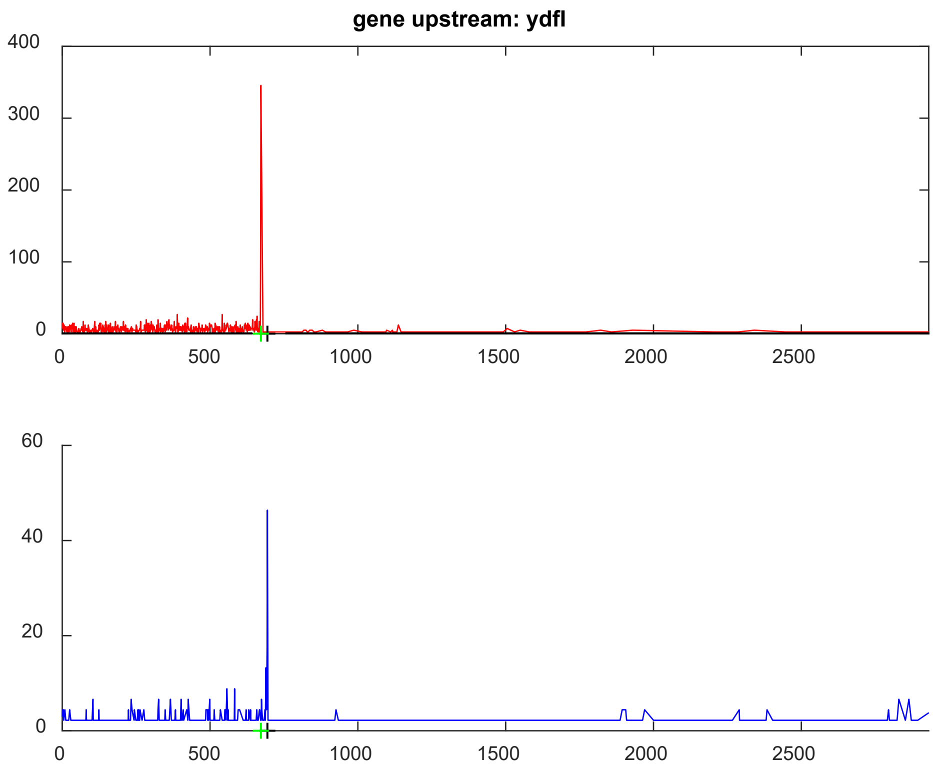
gene upstream: ydfI
400
300
200
100
0
0
500
1000
1500
2000
2500
60
40
20
0
0
500
1000
1500
2000
2500

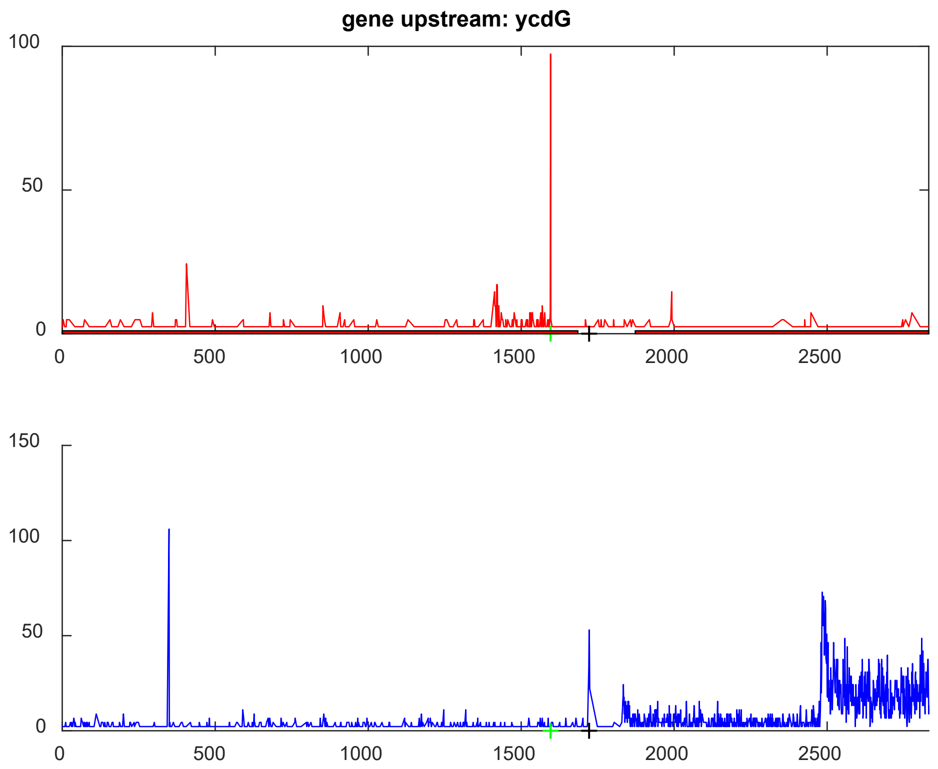

gene upstream: ycdG
100
50
0
0
500
1000
1500
2000
2500
150
100
50
0
0
500
1000
1500
2000
2500

REVIEWERS' COMMENTS:

Reviewer #1 (Remarks to the Author):

I thank the authors for their responses to my questions and critiques. I have responded to their responses in the attached PDF, but fully recommend publication of their work.

Reviewer #2 (Remarks to the Author):

The authors have addressed all of my concerns in the revised manuscript. I now recommend its publication at Nature Communications.
